# Sensing and Filtering Environmental Fluctuations: The Case of Biomolecular Condensates in Plants

**DOI:** 10.1002/advs.75297

**Published:** 2026-04-30

**Authors:** Panagiotis N. Moschou, Dorothee Staiger

**Affiliations:** ^1^ Department of Biology University of Crete Heraklion Greece; ^2^ Institute of Molecular Biology and Biotechnology Foundation for Research and Technology‐Hellas Heraklion Greece; ^3^ Department of Molecular Sciences Uppsala BioCenter Swedish University of Agricultural Sciences, and Linnean Center for Plant Biology Uppsala Sweden; ^4^ RNA Biology and Molecular Physiology, Faculty of Biology Bielefeld University Bielefeld Germany

**Keywords:** biomolecular condensate, light, plant, RNA‐binding proteins, temperature

## Abstract

Biomolecular condensates are membrane‐less assemblies that selectively concentrate proteins, RNAs, and metabolites to integrate developmental and environmental cues. The remarkable diversity of plant condensates reflects the constraints of sessile organisms that must coordinate postembryonic organ development with continuous environmental adaptation. We review how plants employ condensates to integrate temperature, light, redox, and nutrient signals. We provide physicochemical foundations, including phase diagram behavior, critical solution temperature properties, and sticker‐and‐spacer models, as a framework for interpreting how environmental stimuli are transduced into condensate assembly/disassembly. We organize each biological system through a unified scaffold–client–RNA–metabolite framework, distinguishing experimentally validated conclusions from open mechanistic questions. Applying this framework across nuclear, cytoplasmic, chloroplastic, and membrane‐associated condensates, we evaluate how temperature shifts, redox changes, post‐translational modifications, and metabolite fluctuations drive reversible phase transitions. We highlight how saturation concentration thresholds function as nonlinear filters buffering environmental noise, how membrane‐associated phase separation may nucleate cytoplasmic condensates, and where current evidence remains insufficient to distinguish bona fide liquid–liquid phase separation from alternative assembly mechanisms. By grounding plant condensate biology in physicochemical principles and comparative evidential analysis, we identify both well‐supported mechanisms and critical gaps that must be addressed to translate condensate‐biology into strategies for crop‐resilience.

## Graphical abstract

The diversity of plant condensates reflects constraints of sessile organisms to coordinate postembryonic development with environmental adaptation. This review examines how plants employ condensates to integrate temperature, light, redox, and nutrient signals. Grounding plant condensate biology in physicochemical principles and evidential analysis identifies well‐supported mechanisms and critical gaps to be addressed to translate condensate biology into strategies for crop resilience.

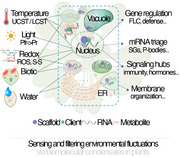

## 1 Introduction

Plants face continuous environmental fluctuations; temperature shifts, changes in light quality and intensity, water availability, and redox perturbations, which demand rapid, reversible cellular responses [1]. These signals are initially transduced at the plasma membrane–cell wall interface, where receptor‐like kinases, mechanosensitive channels, and lipid‐modifying enzymes convert extracellular conditions into intracellular signaling cascades [2]. However, classical signaling models based on linear kinase cascades and transcriptional activation operate on timescales of minutes to hours, whereas many environmental fluctuations require sub‐minute cellular reorganization. Biomolecular condensates, membrane‐less assemblies formed through liquid–liquid phase separation (LLPS), have emerged as a mechanism that bridges this temporal gap, enabling cells to concentrate, sequester, or activate signaling components within seconds through thermodynamically driven phase transitions [3].

The physicochemical environment within plant cells changes rapidly under stress: reactive oxygen species (ROS) accumulate within seconds, cytosolic Ca^2+^ concentration increases transiently, and intracellular pH shifts, all of which directly alter the thermodynamic parameters governing macromolecular phase behavior [3, 4]. ROS, among the earliest signals produced upon stress perception, modify cysteine residues to form disulfide bonds that can promote or dissolve condensates, as demonstrated for the condensates of TERMINATING FLOWER (TMF) and LESION SIMULATING DISEASE 1 (LSD1)/CATALASE 2 (CAT2) (see Sections 5.1 and 5.3). Ca^2+^ transients recruit Ca^2+^‐binding proteins such as CALMODULIN‐LIKE 38 (CML38) into the condensates of stress granules (SGs) [5]. Temperature perturbations alter protein folding equilibria and hydrophobic exposure while cold stress enhances electrostatic interactions (see Section 4). These secondary messengers and physicochemical changes thus provide the molecular inputs that drive the condensate responses detailed in this review.

Condensates differ fundamentally from classical signaling cascades in their assembly logic: rather than sequential enzyme activation, they form through thermodynamically driven phase transitions when scaffold protein concentrations exceed a saturation threshold (*C*_sat_), enabling switch‐like cellular responses within seconds [3, 4]. Their membrane‐free architecture permits rapid, reversible assembly and dissolution without the biosynthetic cost of organelle biogenesis, while their compositional tunability, scaffold proteins, recruited clients, sequestered RNAs, and enriched metabolites, allow a single condensate type to integrate multiple signaling inputs simultaneously. In animal systems, multivalent scaffold–client interactions concentrate kinases, phosphatases, and transcription factors within condensates, dramatically accelerating reaction rates [6]. Whether equivalent organizational principles operate in plant condensates, and how plant‐specific constraints shape them, remains incompletely understood. This review addresses that gap; we first highlight the biological features that make plant condensate systems distinctive (Section 2), then provide concise physicochemical foundations (Section 3) before examining condensate responses to temperature and light (Section 4), redox signals (Section 5), membrane‐associated sensing (Section 6), and nutrient stress (Section 7).

## 2 The Unique Plant Case of Condensates

Plants present a uniquely informative system for condensate biology for three reasons. First, the combination of sessility and postembryonic development means that newly forming organs (i.e., leaves, roots, lateral meristems), must simultaneously establish developmental programs and mount stress responses under ambient conditions, creating a requirement for rapid, reversible compartmentalization [7, 8, 9]. Second, plants experience the full physiological temperature range relevant to both upper critical solution temperature (UCST) and lower critical solution temperature (LCST) phase behavior: as discussed below, cold‐induced condensation of CHLOROPLAST RIBONUCLEOPROTEIN 29A (CP29A) in chloroplasts, FRIGIDA (FRI) in the nucleus and heat‐induced condensation (EARLY FLOWERING 3 (ELF3), SG scaffolds) occur within the same organism across seasons, providing natural comparative systems for dissecting the molecular grammar of thermoresponsive phase separation (Table 1). Third, plant‐specific cellular features, the cell wall–plasma membrane interface, chloroplast and plastid compartments, and unique post‐translational modifications (PTMs), create condensate systems with no direct animal or fungal counterpart, including chloroplastic SGs (SNOWY COTYLEDON 1 (SCO1)/cpSGs), and wall‐sensing condensate nucleation (FERONIA/GLYCINE‐RICH RNA‐BINDING PROTEIN 7 (AtGRP7)) (Table 1). These features make plant condensates not merely a parallel to better‐studied animal systems but a source of mechanistic principles, particularly regarding environmental sensing and noise filtering through saturation concentration thresholds, that are underrepresented in current condensate biology frameworks [9].

**TABLE 1:** Biomolecular condensates formed in response to environmental stimuli in plants. Proteins and systems are organized by the environmental stimulus triggering condensate formation. Scaffold and client assignments, subcellular localization, evidence class, and key references are provided for each system. Evidence class summarizes the types of experimental validation available (see main text for detailed assessment).

Protein/system	Condensate type	Subcellular localization	Scaffold	Clients	Evidence class	References
**Light and Temperature**						
**PhyB**	Photobodies	Nucleus	PhyB (Pfr form); PCH1, RCB, NUCLEAR CONTROL of PEP activity (NCP), HME as co‐scaffolds	PIF1, PIF3 (ubiquitin‐dependent degradation)	In vitro LLPS, in vivo puncta, reversibility, genetics	[18, 19]
**CRY2**	Photobodies	Nucleus	CRY2 (blue light‐dependent oligomerization; scaffold for TCP22/LWD pathway; likely client in m^6^A writer pathway)	TCP22, LWD (circadian); MTA, MTB, FIP37 (m^6^A writers); FIO1/SPA1	In vivo puncta, reversibility, optogenetics	[20, 21]
**ELF3**	ELF3 condensates	Nucleus	ELF3 (PrLD with polyQ; LCST behavior)	ELF4, LUX	In vitro LLPS, FRAP, natural variation (polyQ)	[8]
**FRI**	Transcriptional condensates	Nucleus	FRI (IDR‐driven; UCST behavior)	H3K36me3/H3K4me3 writers (inferred; behavior upon condensation uncharacterized)	In vivo puncta, temperature dependence	[22]
**FCA/FLL2**	Percolation condensates	Nucleus	FCA (tetramer core); FLL2 (co‐scaffold promoting higher‐order assembly)	COOLAIR, FY, FPA, CPSF100, FLD	In vitro LLPS (FCA), single‐particle tracking, genetics	[23, 24, 25]
**GI/FKF1**	Nuclear condensates	Nucleus	GI (dissolution‐based signaling)	FKF1 extracts GI; SVP targeted for degradation	In vivo puncta, temperature‐dependent dissolution	[26]
**DCP5/SSF**	Chromatin‐anchored condensates	Nucleus	DCP5 (IDR‐driven LLPS at FLC locus)	SSF (PrLD‐containing client)	In vitro LLPS, genetics	[27]
**CBF‐SKIP**	Splicing condensates	Nucleus	SKIP (IDR‐mediated)	CBFs, splicing regulators	In vitro LLPS, genetics	[28]
**ALBA4/5/6**	SGs	Cytoplasm	Not scaffolds (SGs form in alba456 mutants); clients	HSF mRNAs (stabilized within SGs)	In vivo co‐localization, genetics, RIP	[29]
**RBGD2/4**	SGs	Cytoplasm	RBGD2/4 (Tyr‐rich LCD‐driven LLPS)	Stress‐responsive mRNAs (HSFA2, HSP70)	In vitro LLPS, FRAP, domain mapping	[30]
**AtGRP7**	GRP7 condensates	Cytoplasm	GRP7 (Tyr residues in G‐rich IDR; FERONIA‐dependent Ser^132^ phosphorylation gates LLPS)	eIF4E1, CSP1/CSP3, mRNAs	In vitro LLPS, FRAP, natural variation (Ser^132^ SNPs)	[31, 32, 33]
**ECT9/ECT1**	m^6^A reader condensates	Cytoplasm	ECT9 (autonomous LLPS via PrLD); ECT1 (conditional, stress‐dependent)	m^6^A‐marked mRNAs, ALBA proteins (via YAIM motif)	In vitro LLPS, genetics, m^6^A‐binding assays	[34, 35, 36]
**SCO1**	cpSGs	Chloroplast	Scaffold unknown (multiple IDR‐containing candidates)	Protein clients: RBPs, HSP90‐5, RABE1b, RH3. Metabolite enrichment: fatty acids, amino acids	In vivo puncta, proteomics, metabolomics	[37]
**CP29A**	CP29A condensates	Chloroplast	CP29A (PrLD‐driven LLPS; UCST behavior)	Predominantly intron‐less chloroplast transcripts; rbcL 5′‐UTR	In vitro LLPS, FRAP, fusion, NMR, genetics	[39, 40]
**STT1/STT2**	STT condensates	Chloroplast	STT1/STT2 heterodimer (C‐terminal IDRs; substrate‐gated)	cpTat substrates: OE23, OE17, PsaN (via WEEPD–RR and LVP‐W motifs)	In vitro reconstitution, domain mapping, genetics	[41, 42]

**Redox and Nuclear Gating**						
**TMF**	Transcriptional condensates	Nucleus	TMF (ALOG domain cysteines C112/C117/C124/C126; H_2_O_2_‐induced disulfide concatenation → IDR‐driven LLPS)	BOP family, TFAM homologs (ALOG domain‐mediated)	In vitro LLPS, redox switching, genetics	[43, 44]
**NUP54/58/62**	NPC (FG‐hydrogel)	Nuclear envelope	FG‐nucleoporins (FG‐repeat IDR‐driven LLPS)	Nuclear transport receptors, cargo	In vitro LLPS, genetics	[45]
**LSD1/CAT2/PEX5**	Ternary redox condensate	Nucleus, peroxisomes, cytosol	LSD1 (scaffold)	CAT2 (via PEX5 obligate bridge)	MST binding, co‐IP, in vivo imaging	[46]

**Membrane association**						
**AP‐3β**	Endomembrane/SG‐associated	TGN, SGs	Disassembly factor (not scaffold); C‐terminal domain for TSN interaction; IDR for membrane association	TSN1/TSN2 (SG docking)	In vivo co‐localization, genetics	[47]
**VAP27‐SCAR/WAVE**	ER–PM contact site condensates	ER–plasma membrane junctions	VAP27 (scaffold at ER–PM contacts)	SCAR/WAVE actin‐regulatory complex	In vivo co‐localization	[48]
**FREE1**	Endosomal condensates	Endosomal membranes (MVBs)	FREE1 (IDR‐driven LLPS at PI3P‐enriched membranes)	ESCRT cargo: PIN2, IRT1, PYR/PYL ABA receptors (via VPS23)	In vitro LLPS, membrane scission assays, genetics	[49]
**SFH8**	Membrane‐associated liquid‐to‐solid condensate	Plasma membrane	SFH8 (SEC14‐like domain; PI lipid‐dependent)	PIN2 (polarity establishment)	In vivo puncta, ESP‐mediated transition	[50]
**RALF‐pectin‐FERONIA‐LLG1**	Extracellular condensates	Cell wall–PM interface	RALF‐pectin (extracellular phase separation)	FERONIA, LLG1 (recruited into condensates; triggers receptor clustering and endocytosis)	In vitro LLPS, in vivo receptor clustering, stress responsiveness	[2]

**Nutrient stress**						
**MED19a/ORE1**	Transcriptional condensates	Nucleus	MED19a (MC‐IDR; acetylation/deacetylation PTM switch; N‐starvation gated)	ORE1 (recruited to activate senescence genes); ELENA1 lncRNA (dissociates complex)	In vivo puncta, FRAP, 1,6‐HD, cross‐species complementation	[51, 52]
**TSN1/TSN2**	SGs	Cytoplasm	TSN1/TSN2 (SN domain‐mediated; IDP scaffold)	SnRK1α1/α2, translation factors (eIF3, eIF4E, eIF4G, 40S), PABPs, TZF1, RH6/8/12, metabolic enzymes	Proteomics, co‐IP, FRET, metabolomics	[53, 54]

**Hypoxia**						
**UBP1C**	SGs	Cytoplasm	UBP1C (3× RRM; TIA‐1/TIAR homologue; energy crisis‐triggered)	Translation initiation factors, PABPs, RH6/8/12, CML38, UBA1a/UBA2a; >1000 mRNAs (U‐rich 3′ UTR)	In vivo puncta, mRNP‐IP, reversibility, genetics	[55]

**FIGURE 1:**
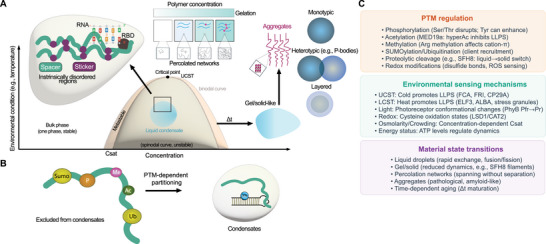
Physico‐chemical principles governing biomolecular condensate formation and regulation in plant cells. (A) Phase behavior, molecular architecture, and material states of biomolecular condensates. The phase diagram (left) plots environmental condition (e.g., temperature) against concentration, showing the transition from a bulk homogeneous phase (one phase) to liquid condensate formation above the saturation concentration (*C*_sat_). The phase diagram depicts two boundaries: the outer binodal (coexistence curve) defining the onset of two‐phase equilibrium, and the inner spinodal curve demarcating the thermodynamically unstable region. Between these boundaries lies the metastable zone, where the homogeneous mixture is thermodynamically unfavorable but kinetically persistent, and phase separation proceeds only through nucleation and growth, requiring stochastic density fluctuations to overcome an energy barrier before discrete droplets form and expand. Within the spinodal boundary, the system is unconditionally unstable: infinitesimal concentration fluctuations amplify spontaneously. At the apex of the phase diagram, the binodal and spinodal curves converge at the critical point, which defines the maximum temperature (for UCST systems; shown here) or minimum temperature (for LCST systems) at which two‐phase coexistence is possible. Inset: intrinsically disordered regions (IDRs) with adhesive ‘sticker’ motifs (purple) connected by flexible ‘spacer’ sequences (green), associated with RNA through an RNA‐binding domain (RBD). Time‐dependent maturation (Δ*t*) drives transitions from liquid condensates toward gel/solid‐like states. Upper center: increasing polymer concentration promotes gelation, percolated network formation, and aggregation, representing distinct material states. Right: condensates exhibit diverse architectures, monotypic (single‐component), heterotypic (multi‐component; e.g., P‐bodies), and layered (compositionally distinct shells). (B) Post‐translational modification (PTM)‐dependent partitioning of proteins into or out of condensates. A protein bearing multiple PTMs, SUMOylation (Sumo), phosphorylation (P), methylation (Me), acetylation (Ac), and ubiquitination (Ub), is shown either excluded from condensates or recruited into membrane‐associated condensates, depending on modification status. (C) Summary of regulatory mechanisms governing plant condensates, organized into three categories. PTM regulation: phosphorylation (Ser/Thr disrupts; Tyr can enhance), acetylation (MED19a: hyperacetylation inhibits LLPS), methylation (Arg methylation affects cation‐π interactions), SUMOylation/ubiquitination (client recruitment), proteolytic cleavage (e.g., SFH8: liquid to solid switch), and redox modifications (disulfide bonds, ROS sensing). Environmental sensing mechanisms: UCST‐cold promotes LLPS (FRI, CP29A); LCST‐heat promotes LLPS (ELF3, RBGD2/4, stress granules); light‐photoreceptor conformational changes (PhyB Pfr to Pr); redox‐cysteine oxidation states (LSD1/CAT2); osmolarity/crowding‐concentration‐dependent *C*_sat_; energy status‐ATP levels regulate dynamics. Material state transitions: liquid droplets (rapid exchange, fusion/fission), gel/solid (reduced dynamics, e.g., SFH8 filaments), percolation networks (spanning without separation), aggregates (pathological, amyloid‐like), and time‐dependent aging (Δ*t* maturation).

**FIGURE 2:**
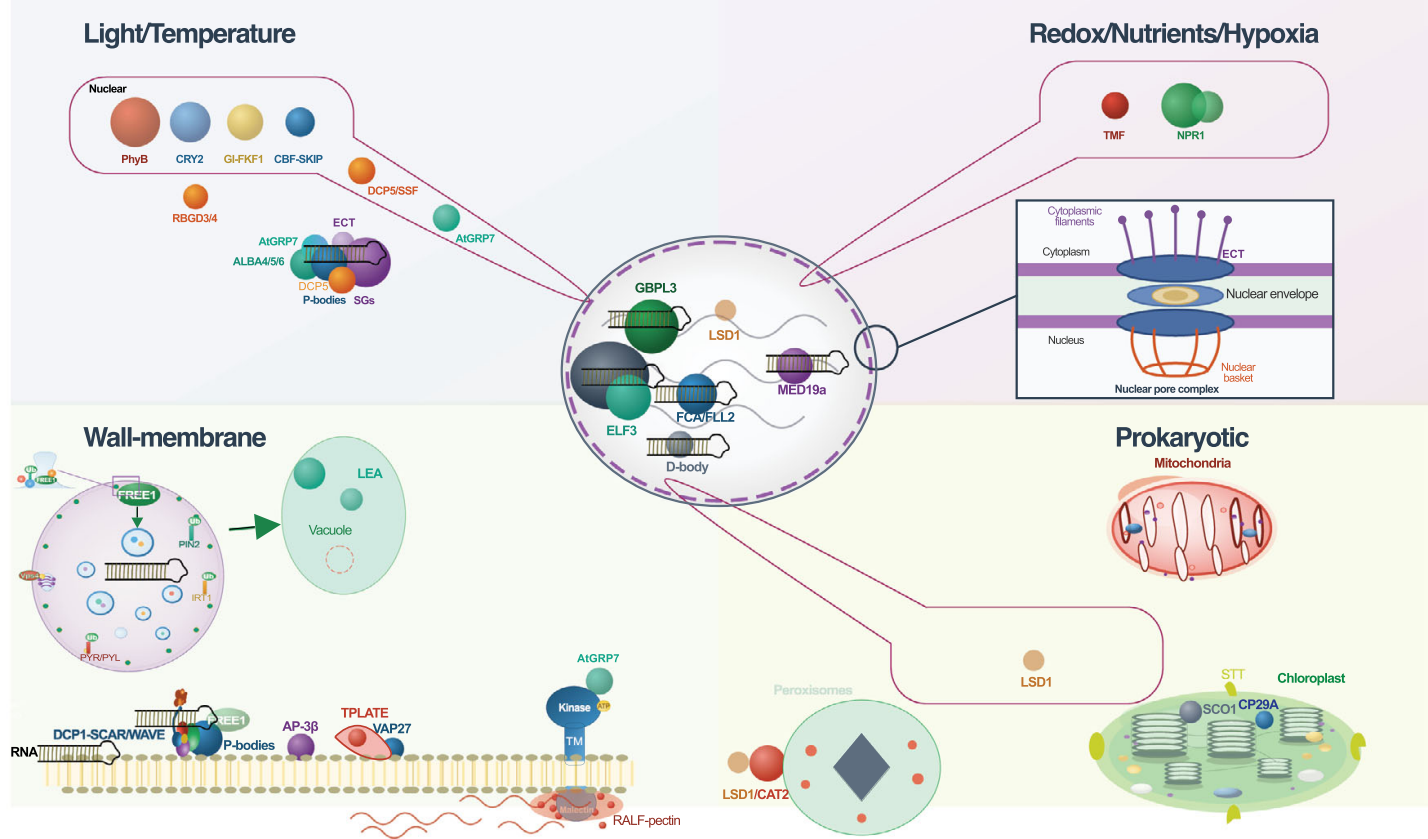
Subcellular organization of biomolecular condensates across plant environmental stress responses. This schematic illustrates the spatial distribution of stress‐responsive condensates across cellular compartments, organized also by the environmental stimuli to which they respond (where relevant). Condensates are depicted in their primary subcellular locations, with proteins functioning as scaffolds, clients, or both indicated. Light/Temperature (upper left). *Nuclear*: PhyB photobodies (Pr→Pfr‐dependent, ∼1 µm foci recruiting PIFs); CRY2 blue light‐induced condensates (FAD‐mediated, co‐condensing with m^6^A methyltransferase components MTA, MTB, FIP37 and SPA1); GI‐FKF1 temperature‐dependent flowering condensates; CBF‐SKIP cold‐induced splicing condensates (CBF IDRs facilitate SKIP LLPS); DCP5/SSF condensates at *FLC* chromatin (DCP5 scaffold, SSF PrLD client). *Cytoplasmic*: P‐bodies and stress granules (SGs) as mRNA triage centers; RBGD2/4 (tyrosine‐rich IDRs, heat‐induced SG scaffolds); ALBA4/5/6 (ALBA domains + RGG motifs, heat‐protective granules); DCP5 (P‐body‐localized, chromatin‐relocalized in DCP5/SSF); AtGRP7 (RRM + glycine‐rich IDR, FERONIA‐phosphorylated); ECT m^6^A readers (YTH domains, *π–π* stacking‐dependent thermomorphogenic condensates). Redox/Nutrients/Hypoxia (upper right). *Nuclear*: TMF (H_2_O_2_‐induced disulfide condensates); NPR1 SINCs (redox‐switched, nuclear transcriptional hubs + cytoplasmic E3 ligase platforms). *Nuclear pore complex*: FG‐Nups (NUP62, temperature‐sensitive *π–π* hydrogel barrier). Nucleus (central). GBPL3 defense‐activated condensates (GDACs: chromatin‐modifying proteins, Pol II, temperature‐sensitive at 28°C); ELF3 LCST‐driven condensates (polyQ PrLD, sequestering evening complex); MED19a‐ORE1 heterotypic transcriptional condensates (nitrogen starvation); FCA/FLL2 condensates (*COOLAIR* processing); D‐bodies (SE scaffold for DCL1, HYL1, CYP71, HEN1; SAM‐supported miRNA methylation; [70, 73] not discussed further). Wall–Membrane (lower left). *Endosomal*: FREE1 (FYVE domain–PI3P binding, IDR‐driven LLPS on MVBs, ESCRT‐I/VPS23 interaction, trafficking of PIN2, IRT1, PYR/PYL). *Late embryogenesis abundant (LEAs)*: LEA proteins in the vacuole (desiccation‐protective scaffolds [72]). *Plasma membrane*: DCP1‐SCAR/WAVE [150] (condensate‐membrane contacts); AP‐3β (19S proteasome recruitment for SG disassembly); VAP27 (ER‐PM contact site condensates); TPLATE/AtEH/Pan1 (condensate‐driven clathrin‐coated pit assembly). *Cell wall*: RALF‐pectin extracellular LLPS recruiting FERONIA receptor complexes. Prokaryotic‐type organelles (lower right). *Chloroplast*: STT1/STT2 (ankyrin‐repeat scaffolds, substrate‐induced concentration for cpTat sorting); SCO1 cpSGs (33°C heat‐induced, enriched in RBPs, HSP90‐5, RuBisCO activase); CP29A UCST‐type cold condensates (PrLD between two RRMs, nucleoid‐proximal). *Mitochondria*: RNA granule systems uncharacterized in plants. *Peroxisomes*: LSD1/CAT2/PEX5 layered ternary complexes with redox‐tunable compartmentalization.

Here, we discuss five types of condensates according to the input signal that leads to their formation, covering a large scale of stimuli in plant responses (Table 1). We organize each condensate in (i) scaffolding molecules, and, where identified, (ii) client protein and nucleic acid molecules, (iii) metabolite molecules, and (iv) PTMs (Figure 1 for condensate formation principles). Throughout, we explicitly distinguish experimentally validated conclusions from interpretive extensions and open questions. We also focus on how environmental stimuli are converted to physicochemical inputs at the molecular level, how they are converted to signaling outputs, and how they can affect the above four organizational components. While condensates play critical roles in plant immunity, we do not systematically cover biotic stress‐responsive condensates; for comprehensive treatment of immunity‐related condensates, we refer readers to recent excellent reviews [10, 11, 12, 13]. Furthermore, our coverage reflects deliberate selection of examples that best illuminate mechanistic principles rather than proportional representation of the existing literature. Select immunity‐related condensates (e.g., NON‐EXPRESSOR OF PR GENES 1 (NPR1) and SINCs [14], GUANYLATE‐BINDING PROTEIN LIKE 3 (GBPL3)/GDACs [15], NUCLEAR PORE COMPLEX (NPC) components) appear in Figure 2 and Table 1 as contextual examples where they illuminate general physicochemical principles, rather than as systematically treated case studies.

## 3 An Overview of Condensate Formation at a Macroscale

Condensates form when a uniform mixture of biomolecules spontaneously separates into coexisting dense and dilute phases, driven by free energy minimization [16] (Figure 1A). The Flory–Huggins theory provides a foundational framework for this process, modelling phase separation as a competition between mixing entropy (Δ*S*_mix_), which favors homogeneous distribution, and interaction enthalpy (Δ*H*_mix_), which can favor demixing when intermolecular contacts, hydrogen bonds, electrostatic attractions, hydrophobic effects, exceed a critical strength [17]. This balance is captured by the interaction parameter *χ*: when *χ* exceeds a critical value that depends on polymer chain length and volume fraction, the system demixes into polymer‐rich condensates and polymer‐poor phases. For typical biopolymers with hundreds to thousands of residues, this critical *χ* is relatively small (∼0.5). Note that effective *χ* for real proteins depends on sequence‐specific interactions that Flory–Huggins averages over. Nevertheless, the low χ explains why biopolymers with many residues can phase‐separate through relatively weak per‐residue interactions, and why cells’ interiors and corresponding polymers operating near the phase boundary can be tipped between mixed and demixed states by modest environmental changes.

The phase diagram reveals the boundaries governing this behavior [3, 56, 57]. The binodal (coexistence) curve separates single‐phase from two‐phase regions; inside it, the spinodal curve delineates regions of absolute thermodynamic instability where separation occurs spontaneously (Figure 1A). Between binodal and spinodal lies the metastable region, where nucleation events must overcome a free energy barrier before phase separation proceeds. For biological condensates, the saturation concentration (*C*_sat_) typically ranges from sub‐micromolar to tens of micromolar depending on valency and interaction strengths; for plant proteins, *C*_sat_ values remain largely unmeasured. Critically, the binodal acts as the threshold of a nonlinear biological filter. Sub‐threshold fluctuations in scaffold concentration or temperature fail to nucleate condensates, and brief excursions into the metastable region beyond the binodal are further damped by nucleation barriers; only sustained stimuli produce assembly. Above the binodal, the dilute‐phase concentration is buffered to Csat, providing a complementary form of fluctuation control: assembly is sharp, but free‐monomer levels are robust to overshoot. This threshold behavior allows sessile organisms to discriminate genuine stress signals from stochastic noise [58].

Temperature determines which side of the phase boundary a system occupies. UCST systems undergo phase separation upon cooling, as reduced thermal energy strengthens enthalpic interactions such as hydrogen bonds and electrostatic attractions [59, 60, 61, 62]. LCST systems separate upon heating, as elevated temperatures increase the entropic cost of maintaining hydration shells around hydrophobic residues, favoring desolvation and assembly [59, 60, 61, 62]. Many plant condensates exhibit LCST behavior, such as heat shock proteins, SG scaffolds, and photobody components which condense during heat stress. Yet, fewer documented cases in plants display UCST behavior (e.g., CP29A, and FRI under cold). Whether this asymmetry reflects a genuine compositional bias in plant proteins, such as the enrichment of aliphatic and aromatic residues in thermoresponsive domains recently reported by [63], or observational bias toward agriculturally relevant heat stress, remains unresolved.

The kinetics of phase separation follow two distinct pathways described by the Cahn–Hilliard framework [64]. Spinodal decomposition occurs deep within the unstable region, producing spontaneous, spatially continuous separation into bicontinuous network structures. Nucleation‐and‐growth operates in the metastable region, producing discrete droplets that coarsen through coalescence and Ostwald ripening. In principle, biological condensates could employ both pathways: rapid stress onset favors spinodal decomposition, while gradual environmental changes induce nucleation‐and‐growth, though direct observation of spinodal decomposition in vivo remains limited.

At the molecular level, the sticker‐and‐spacer model explains how intrinsically disordered regions (IDRs), protein segments that lack stable tertiary structure and instead sample a dynamic conformational ensemble, drive condensate properties through their sequence composition [65]. Adhesive ‘stickers’, aromatic residues, RGG motifs, charged patches, mediate the multivalent intermolecular contacts that drive phase separation, while intervening ‘spacers’ control sticker density, accessibility, and the entropic cost of chain collapse. This framework explains why identical sticker types with different spacer arrangements yield condensates with distinct viscosities, exchange kinetics, and client specificities. It also clarifies how PTMs regulate condensates: phosphorylation of stickers can eliminate adhesiveness, while modifications of spacers alter flexibility [66, 67]. Multivalency, the number of interaction sites per molecule, is the key parameter: each additional sticker exponentially increases interaction network complexity, dramatically lowering *C*_sat_ and explaining why modest changes in expression levels or PTM states can trigger abrupt condensate formation or dissolution.

Not all condensates are dense. Some ‘inverse’ condensates display lower bulk density than the surrounding cytoplasm, arising from excluded volume effects, where large scaffold molecules create solvent‐accessible voids, or from specific interaction geometries in percolated networks that trap substantial water within their architectures [68]. In such assemblies, protein scaffolds form airy, interconnected meshworks rather than compact liquid droplets, conceptually analogous to foam structures where the biopolymer phase occupies less volume fraction than the surrounding aqueous milieu. This architecture has important functional implications. Within percolated networks, molecules alternate between transient confinement at crosslink nodes and diffusion‐like motion through solvent‐filled channels, yielding effective diffusion coefficients intermediate between those of liquid droplets (where viscosity limits mobility) and the dilute cytoplasm [69]. Such intermediate exchange dynamics could sustain metabolic flux by permitting substrates and products to traverse the condensate without the diffusion penalties imposed by the high macromolecular crowding of dense phase‐separated droplets, while still concentrating enzymes above their effective *K*_m_ through scaffold‐mediated tethering. The ability to maintain both spatial organization and rapid molecular exchange makes low‐density networks particularly attractive as functional platforms in metabolically active compartments. The chloroplast stroma, where RuBisCO, RuBisCO activase, and translation machinery operate at high local concentrations under photosynthetically active conditions, represents a compartment where such architectures could reconcile the competing demands of enzyme concentration with the requirement for rapid CO_2_/metabolite diffusion. However, direct rheological or single‐molecule tracking measurements that would quantify diffusion coefficients, mesh size, or percolation thresholds within plant inverse condensates are currently lacking, and the functional advantages proposed here remain inferred from polymer physics principles rather than demonstrated experimentally.

Rather than existing solely as passive thermodynamic equilibria, many condensates are actively maintained as non‐equilibrium structures. ATP‐consuming processes, chaperone‐mediated remodeling, kinase‐driven modification of interaction surfaces, RNA helicase unwinding of secondary structures, continuously control condensate composition and prevent maturation into pathological solid‐like aggregates. The adenylate energy charge (AEC) serves as a cellular energy sensor that directly influences condensate stability, with ATP depletion often triggering protective condensate formation [74, 75]. Myosin‐powered active transport along actin filaments provides an additional regulatory mechanism [76]: AUXIN RESPONSE FACTOR 7 (ARF7) and ARF19 form cytoplasmic condensates in Arabidopsis through PB1 domain polymerization and intrinsically disordered middle region interactions, sequestering these auxin‐responsive transcription factors from nuclear targets [77]. ARF19 condensates exhibit rapid directional movement (∼10 µm/s) along myosin XI‐driven actin tracks, and disruption of actin polymerization with latrunculin B reduces condensate size, demonstrating that cytoskeletal forces enhance assembly by increasing effective local concentrations and encounter rates, thereby lowering *C*_sat_. Whether this mechanism operates in other motile plant condensates remains to be determined.

### 3.1 Alternative Assembly Beyond Classical Phase Separation

Biological condensates are not limited to LLPS. Several alternative assembly mechanisms generate structures with distinct material properties matched to specific functional requirements (Figure 1A) [58]. Percolation produces system‐spanning networks when molecular concentrations exceed a critical percolation threshold (*C*_perc_), yielding structures that lack sharp phase boundaries and exhibit intermediate properties between solutions and gels with molecular mobility combined with mechanical persistence [57]. Percolation can occur independently of phase separation or within phase‐separated droplets, yielding distinct material properties in each case. This mechanism is particularly relevant to nuclear pore complex assembly in plants, where FG‐nucleoporin networks form selective hydrogel barriers (Section 5.2). Gelation transforms condensates into elastic solid‐like structures through extensive intermolecular crosslinking, creating networks that resist deformation and cytoplasmic forces [78, 79]. Classical gelation has not been directly demonstrated for plant condensates, although it has been implied [58].

Aggregation produces kinetically trapped, essentially irreversible structures with minimal internal molecular exchange [80]. While often pathological, plants exploit controlled aggregation for adaptive purposes. Seed storage proteins form highly compacted amyloid‐like aggregates during desiccation that disassemble during germination [71], and the prion‐like protein FLOE1 undergoes hydration‐dependent phase separation that regulates germination timing [81, 82]. Proteins with a subclass of IDR, the prion‐like domains (PrLDs), are widespread in plant proteomes, nearly 500 PrLD‐containing proteins were identified in Arabidopsis alone [83], and computational network analysis across 39 plant genomes has revealed their enrichment in stress‐responsive and memory‐associated regulatory networks [84]. However, while several plant PrLD proteins undergo phase separation in vivo, bona fide prion‐like conformational self‐propagation has only been demonstrated for the LUMINIDEPENDENS PrD in a heterologous yeast system [83], and no such propagation has been confirmed in planta. Furthermore, whether bona fide self‐templating prion propagation, the cross‐generational transmission of conformational states through protein‐based inheritance, occurs in plants remains experimentally undemonstrated.

These assembly mechanisms exist on a continuum of exchange dynamics and mechanical stability, from LLPS droplets (liquid‐like molecular exchange: seconds) through percolation networks (minutes to hours) and gels (elastic, deformation‐resistant) to aggregates (solid‐like; hours to irreversible), and individual condensate systems can transition between states through PTMs, chaperone activity, and environmental cues [58, 85].

### 3.2 Modulators of Condensation at the Atomic/Molecular Level

IDRs, typically 30–200 residues long, are the principal molecular drivers of condensate assembly [86, 87]. Their conformational flexibility enables multivalent contacts that folded domains cannot readily achieve, and their sequence composition directly determines condensate properties (Figure 1A). Several functionally distinct IDR classes are relevant to plant condensate systems discussed in this review.

The above mentioned PrLDs are enriched in Q, N, G, and S residues and drive homotypic interactions through weak, aggregation‐prone contacts that promote scaffold assembly. In plants, PrLDs mediate temperature‐dependent condensation of EARLY FLOWERING 3 (ELF3; polyQ‐type, LCST behavior; Section 4.1.3) and cold‐induced assembly of CP29A (Section 4.3.2). Polyampholytic IDRs contain interspersed positive (R, K) and negative (D, E) charges whose spacing pattern‐rather than net charge‐determines phase separation propensity [88]. These regions are sensitive to ionic strength and pH, enabling environmental responsiveness; the mixed‐charge IDR (MC‐IDR) of MEDIATOR SUBUNIT 19a (MED19a) exemplifies this class, driving nitrogen starvation‐responsive condensation through electrostatic interactions modulated by lysine acetylation (Section 7.1). Aromatic‐rich IDRs mediate phase separation through π–π stacking and cation–π interactions. FG‐repeat domains in nucleoporins create the hydrogel barrier of the nuclear pore complex through phenylalanine‐driven aromatic contacts [89], and tyrosine‐rich low‐complexity domains in RNA‐binding GLYCINE‐RICH DOMAIN 2/4 (RBGD2/4) drive heat‐induced SG formation (Section 4.2.2). RGG/RG‐rich motifs, predominantly found in RNA‐binding proteins, mediate RNA recruitment into condensates; ALBA4/5/6 proteins use C‐terminal RGG motifs to sequester heat shock factor mRNAs (Section 4.2.1). Arginine methylation within these regions modulates RNA binding affinity and phase separation propensity [13, 90, 91, 92, 93]. Phosphorylation‐sensitive IDRs containing S/T residues serve as regulatory switches, exemplified by FERONIA‐mediated Ser^132^ phosphorylation of AtGRP7's G‐rich IDR controlling temperature‐dependent condensation (Section 4.2.3).

Many plant stress‐responsive proteins contain combinations of these IDR classes within a single polypeptide [94, 95], creating multivalent interaction networks where distinct biophysical mechanisms, electrostatic, aromatic, hydrogen‐bonding, respond to different environmental inputs, enabling compositionally complex condensates that integrate multiple signals simultaneously.

### 3.3 Condensates Are Not Only Regulated by Disorder

Phase separation is not an inherent property of disorder; many IDRs remain chemically inert under basal conditions, with their interaction potential activated only by specific cues such as oxidation, phosphorylation, or binding partners [96, 97] (Figure 1B,C). Conversely, structured domains can drive phase separation independently of IDRs: the ARF7/ARF19 PB1 polymerization domain nucleates cytoplasmic condensates through oligomeric interactions [77], and the FLOWERING TIME CONTROL PROTEIN A (FCA) RNA recognition motif is dispensable for core tetramer formation but required for higher‐order multimerization into macromolecular condensates (Section 4.1.4). These examples establish that plant condensate assembly integrates contributions from both disordered and folded regions, with their relative importance varying by system.

PTMs serve as the primary regulatory switches controlling condensate phase boundaries (Figure 1C). In plants, several PTM mechanisms have been directly linked to condensate dynamics. Phosphorylation exerts context‐dependent effects: FERONIA‐mediated Ser^132^ phosphorylation of AtGRP7 controls temperature‐dependent condensation (Section 4.2.3), while S/T phosphorylation more generally disrupts condensates through electrostatic repulsion and Y phosphorylation can enhance π–π interactions [98, 99]. Lysine deacetylation of MED19a under nitrogen starvation removes an inhibitory constraint, enabling LLPS through its mixed‐charge IDR (Section 7.1). Arginine methylation modulates cation–π interactions in RNA‐binding protein condensates [100, 101]. Cysteine oxidation drives disulfide bond‐dependent condensation of TMF under H_2_O_2_ accumulation (Section 5.1) and controls the redox‐tunable LSD1/CAT2 compartmentalization system (Section 5.3). Proteolytic cleavage has emerged as a particularly powerful condensation switch in plants: separase‐mediated cleavage of SFH8 at the *φ*EXXR motif converts dynamic membrane‐associated liquid clusters into stable filamentous structures, demonstrating that irreversible PTMs can enforce permanent material state transitions for developmental patterning [50, 102].

## 4 Light and Temperature Crosstalk With Condensates

Light and temperature converge on condensate biology through two distinct physicochemical mechanisms. First, photoreceptor conformational switching, the interconversion of PHYTOCHROME (Phy) B and the flavin adenine dinucleotide (FAD)‐mediated photocycle of cryptochrome 2 (CRY2), directly alters protein interaction surfaces that drive phase separation, creating condensates whose assembly is light‐reversible. Second, temperature acts on the intrinsic phase behavior of scaffold proteins: LCST‐driven condensation during heat stress (e.g., ELF3, SG scaffolds) and UCST‐driven assembly under cold (CP29A, FRI) reflect distinct thermodynamic mechanisms operating through hydrophobic exposure and electrostatic enhancement, respectively [103, 104]. Critically, these two inputs are not independent, as phytochrome thermal reversion couples photoreceptor state to ambient temperature, enabling photobodies to function as integrative sensors of both light and thermal environment (Section 4.1.1). We organize the following case studies by subcellular localization: nuclear condensates (Sections 4.1.1–4.1.4), cytoplasmic assemblies (Section 4.2), and chloroplastic condensates (Section 4.3). Figure 2 provides a spatial overview of these systems and Table 1 summarizes their molecular components and evidence classes.

### 4.1 Nuclear Condensates

#### 4.1.1 PhyB Photobodies

Phys are red/far‐red light receptors encoded by five genes (*PHYA–E*) in dicotyledonous species. The photoreversible Pr/Pfr interconversion, where Pr absorbs at ∼660 nm (red) to form Pfr and reverts upon ∼730 nm absorption (far‐red), constitutes the primary light switch governing photomorphogenic development [105]. This photoreversibility has been directly linked to condensate formation. Chen et al. (2022) demonstrated that PhyB undergoes LLPS in vivo and in vitro in a light‐ and temperature‐dependent manner, establishing photobodies as bona fide phase‐separated condensates rather than mere aggregates [18]. The active Pfr form of PhyB is the primary scaffold for photobody assembly. PhyB contains an N‐terminal photosensory domain with a covalently bound tetrapyrrole chromophore and a disordered N‐terminal extension (NTE) that promotes photobody localization [18, 106]. Upon red light activation, Pfr translocates to the nucleus and assembles into ∼1 µm foci termed photobodies [19, 107, 108, 109]. Several phytochrome‐interacting proteins, photoperiodic control of HYPOCOTYL LENGTH 1 (PCH1), regulator of chloroplast biogenesis, nuclear control of PEP activity, and HEMERA (HME), are additionally required for photobody formation and may function as co‐scaffolds [110]. HME, structurally similar to the yeast ubiquitin‐binding protein RADIATION‐SENSITIVE 23 (RAD23), is dually localized between the chloroplast and the nucleus [111]; Pfr stabilizes HME in the nucleus to promote photobody assembly and subsequent degradation of PHYTOCHROME INTERACTING FACTOR (PIF1) and PIF3 [112].

Furthermore, Pfr undergoes thermal reversion to Pr at elevated temperatures independently of light, with increasing temperature (12°C–27°C) progressively redistributing PhyB‐GFP from large photobodies to small foci and the nucleoplasm [113, 114, 115]. PCH1 protects Pfr from thermal reversion, functioning as a temperature‐sensitivity modulator [116]. Plants lacking photobodies show increased hypocotyl elongation responsiveness to temperature [116], and not all photobody populations disassemble equivalently upon warming, suggesting subpopulations with distinct thermal sensitivity [115]. Whether these differential sensitivities reflect distinct material properties (e.g., viscosity, crosslinking density) remains untested, as no biophysical measurements of material states across photobody subpopulations have been reported. If confirmed, this would suggest that photobody subpopulations with distinct material properties enable graded rather than binary responses to temperature, adding an additional layer of signal processing beyond the threshold behavior inherent to phase separation.

PIFs are recruited into photobodies as clients, where they undergo ubiquitination and proteasomal degradation, relieving transcriptional repression of photomorphogenic programs. PhyB simultaneously disrupts the CONSTITUTIVE PHOTOMORPHOGENIC 1/SUPPRESSOR OF PHYA‐105 1 (COP1/SPA1) E3 ligase complex, stabilizing the transcription factor ELONGATED HYPOCOTYL 5 (HY5) to activate light‐responsive gene expression [108]. No RNA components have been identified within photobodies, and direct metabolite enrichment (e.g., phytohormones) has not been demonstrated. Whether photobodies concentrate specific mRNAs or small‐molecule signals remains an open question.

#### 4.1.2 CRY2 Photobodies

The blue light receptor CRY2 comprises an N‐terminal photolyase homologous region (PHR) that binds the FAD chromophore and a C‐terminal extension (CCE) domain. Blue light irradiation triggers CRY2 oligomerization and formation of nuclear speckles (equivalent to photobodies), observed both for CRY2‐GFP fusions [118] and endogenous protein by immunostaining [119], with rapidly reversible assembly kinetics [20]. Whether CRY2 functions as a primary scaffold or as a client recruited into condensates nucleated by interaction partners (see below) may depend on the specific condensate context, a distinction that has not been formally resolved.

In one pathway, CRY2 photobodies recruit the transcriptional regulators TEOSINTE BRANCHED 1 /CYCLOIDEA/PCF 22/LIGHT‐REGULATED WD (TCP22 and LWD) to the *Circadian* *clock* *associated* *1* (*CCA1*) promoter, activating circadian clock gene transcription consistent with the long‐period clock phenotype of *cry1 cry2* mutants [21]. In a parallel pathway, photoactivated CRY2 co‐condenses with subunits of the N^6^‐methyladenosine (m^6^A) writer complex, the most prevalent internal modification of eukaryotic mRNA, which regulates transcript stability, splicing, and translation [129]. These subunits include the catalytic methyltransferase MTA (orthologue of mammalian METTL3), the catalytically inactive MTB (orthologue of METTL14) that functions as an RNA‐binding scaffold positioning substrates for MTA‐mediated methylation, and the regulatory subunit FKBP12 INTERACTING PROTEIN 37  (FIP37), which is required for efficient m^6^A deposition; in these co‐condensates, CRY2 likely functions as a client rather than scaffold [20, 120]. CRY2 additionally interacts indirectly with the methyltransferase FIONA1 (FIO1, orthologue of METTL16) through Suppressor of Phytochrome A1 (SPA1)‐mediated co‐condensation; CRY2 oligomerization increases its SPA1 affinity, and SPA1/CRY2 activate FIO1 methylase activity in vitro [121]. Whether MTA‐ and FIO1‐containing CRY2 condensates represent the same or distinct assemblies remains unresolved.

CRY2 condensates regulate RNA through epitranscriptomic modification rather than direct sequestration. Light‐induced CRY2/MTA co‐condensation promotes m^6^A modification of *CCA1* mRNA, enhancing its stability, an effect reduced by the condensation inhibitor 1,6‐hexanediol, though this inhibitor's non‐specificity limits its interpretive value as sole evidence for LLPS dependence [20]. FIO1‐dependent m^6^A modification targets a broader transcript pool including chlorophyll homeostasis mRNAs, whose increased translation is consistent with the pale green *fio1* phenotype [121]. These m^6^A‐modified transcripts are subsequently interpreted by EVOLUTIONARILY CONSERVED C‐TERMINAL  (ECT) reader proteins that themselves form condensates (Section 4.2.4), establishing a multi‐step condensate‐to‐condensate regulatory cascade. No metabolite enrichment within CRY2 photobodies has been reported. FAD serves as the chromophore cofactor essential for photocycling but whether it functions as an enriched metabolite within the condensate phase is unknown.

#### 4.1.3 ELF3 as Thermosensor

ELF3 contains a PrLD with polyglutamine (polyQ) repeats that undergoes LCST‐driven phase separation at elevated temperatures [8]. Jung et al. (2020) demonstrated that purified ELF3‐PrLD undergoes reversible condensation in vitro with a temperature‐dependent phase boundary, and that ELF3 forms nuclear condensates in vivo at warm temperatures. The polyQ tract length is the critical molecular determinant: Arabidopsis accessions with longer polyQ tracts exhibit lower critical temperatures for condensation and correspondingly stronger thermal responsiveness, providing a direct molecular basis for natural variation in thermosensing across geographical populations [8]. This establishes ELF3 as the clearest example of a plant protein whose IDR sequence composition directly programs thermoresponsive phase behavior, a principle recently generalized by the finding that aliphatic and aromatic enrichment in PrLDs predicts heat‐induced condensation across diverse proteins [63] (see Perspective).

At permissive temperatures, ELF3 functions within the evening complex of the circadian clock, interacting with ELF4 and the transcription factor LUX ARRHYTHMO (LUX) to repress clock‐regulated target genes including *PIF4* [122]. ELF4 binding modulates ELF3 condensation behavior: interaction with ELF4 promotes ELF3 nuclear speckle formation [123], suggesting that client interactions can tune scaffold phase boundaries. Upon LCST‐driven condensation at elevated temperatures, ELF3 is sequestered from target promoters, derepressing *PIF4* transcription and enabling thermomorphogenic growth responses [8]. This mechanism constitutes a direct thermosensory switch where condensate formation and not merely conformational change, removes a transcriptional repressor from its targets, converting a continuous temperature input into a nonlinear transcriptional output. No specific RNA components have been identified within ELF3 condensates, and metabolite enrichment has not been characterized. Whether the evening complex target transcripts are physically associated with or excluded from ELF3 condensates remains an open question with mechanistic implications: sequestration of target mRNAs within condensates would add a post‐transcriptional layer to the derepression mechanism, whereas exclusion would limit ELF3's role to transcriptional control.

#### 4.1.4 Temperature‐Dependent Flowering Regulation Through FLC

The *FLOWERING LOCUS C* (*FLC*) locus is regulated by at least four distinct condensate systems, each operating through different mechanistic logic, making it the most condensate‐dense regulatory node described in plant biology so far.

FRI condensates (UCST‐type scaffold): The intrinsically disordered protein FRIGIDA (FRI) activates *FLC* transcription at warm temperatures by recruiting H3K36me3 and H3K4me3 methyltransferases to the *FLC* promoter [22]. Upon cold exposure, FRI undergoes phase separation into nuclear condensates, consistent with UCST behavior, sequestering it away from the promoter and repressing *FLC* transcription. FRI condensation is alleviated at higher temperatures, preventing premature flowering during autumn temperature fluctuations [22]. Histone methyltransferases as clients are recruited to FRI at the promoter but their behavior upon FRI condensation is uncharacterized. No RNA or metabolites have been reported for this system.

FCA/FLL2 condensates (hierarchical percolation scaffold): The plant‐specific RNA‐binding protein FCA interacts with the coiled‐coil protein FIO1‐LIKE 2 (FLL2) to form liquid‐like condensates that concentrate 3’‐end processing machinery at the *FLC* locus [23, 24]. Single‐particle tracking of FCA‐mScarlet‐I in vivo revealed a modular assembly hierarchy: a core FCA tetramer forms independently of RNA binding, then multimerizes into higher‐order particles that coalesce into macromolecular condensates via FLL2 [25]. A mutation in the FCA RNA recognition motif disrupted multimerization and condensate formation but not core tetramer assembly, establishing that scaffold oligomerization and RNA‐dependent higher‐order assembly are separable steps, a finding that directly parallels the percolation‐to‐condensation transition described in Section 3.1.

Regarding RNA enrichment, the antisense long non‐coding RNA *COOLAIR*, transcribed from downstream of *FLC*, forms an R‐loop at the locus. FCA/FLL2 condensates promote proximal polyadenylation of *COOLAIR*, releasing the R‐loop and enabling recruitment of the histone demethylase Flowering locus D (FLD), which removes H3K4me1 to silence *FLC* transcription [23, 24]. *COOLAIR* thus functions as both a regulatory RNA substrate and a structural component that nucleates condensate–chromatin interactions. No metabolites have been reported for this system.

GI/FKF1 condensates (temperature‐dependent dissolution): Gigantea (GI) forms liquid‐like nuclear condensates at low ambient temperature. With increasing temperature, the F‐box protein flavin‐binding KELCH Repeat F‐box 1 (FKF1) extracts GI from condensates; the resulting GI‐FKF1 complex targets the floral repressor short vegetative phase (SVP) for proteasomal degradation, derepressing *FLOWERING LOCUS T* (*FT*) and accelerating flowering [26]. This system represents an inverted logic relative to FRI: temperature dissolves rather than promotes condensation, with dissolution rather than assembly constituting the active signaling output. No RNA or metabolites have been reported for this system.

DCP5/SSF condensates (chromatin‐anchored scaffold): The component of the cytoplasmic condensates known as ‘P‐bodies’, decapping protein 5 (DCP5), escapes from P‐bodies and nucleates LLPS through its IDRs at the *FLC* locus. At the *FLC* locus, SSF acts as the chromatin‐anchoring scaffold and recruits DCP5; the resulting SSF–DCP5 condensate represses FLC. DCP5 enhances SSF LLPS in trans through PrD–PrD interactions [27]. DCP5/SSF condensates anchor to *FLC* chromatin through interactions with nascent transcripts and DNA regulatory elements, providing locus specificity.

Nucleic acid interactions are required for chromatin anchoring, but whether specific non‐coding RNAs are enriched within DCP5/SSF condensates remains undemonstrated. Furthermore, no metabolites have been reported for this system. An unresolved question is whether DCP5 recruitment to nuclear *FLC* condensates competes with its canonical cytoplasmic P‐body function, creating a potential regulatory trade‐off between post‐transcriptional mRNA processing and vernalization‐mediated transcriptional memory, though partitioning dynamics between these compartments have not been measured.

#### 4.1.5 CBF‐SKIP Complex in Cold Stress

Under cold stress, accumulated C‐REPEAT BINDING FACTORS (CBFs) interact with SKI‐INTERACTING PROTEIN (SKIP), a spliceosome‐associated protein, through IDRs present in both partners, promoting formation of nuclear condensates at actively transcribed cold‐responsive loci [28]. SKIP appears to function as the primary structural scaffold of the condensate, with CBF accumulation triggering its assembly through IDR‐mediated interaction. CBFs provide chromatin targeting through their C‐repeat/dehydration‐responsive element (CRT/DRE) promoter‐binding activity. The condensates recruit splicing regulators including serine/arginine‐rich (SR) proteins and heterogeneous nuclear ribonucleoproteins (hnRNPs) as clients, locally concentrating splicing machinery at sites of nascent cold‐responsive pre‐mRNA production [28]. This spatial coupling of transcription and splicing within a single condensate has been proposed to shift splice site selection by elevating local splicing factor concentrations, generating cold‐adapted protein isoforms that enhance freezing tolerance [28, 124].

Cold‐regulated transcripts serve as both splicing substrates and potential structural components of the condensate through multivalent RNA–protein interactions [28]. However, whether specific RNAs are required for condensate integrity, as opposed to being passively processed within pre‐formed condensates, has not been directly tested through RNA depletion experiments. So far, no metabolites have been reported for this condensate.

Furthermore, the CBF–SKIP condensate model derives primarily from a single study [28]. Key claims requiring independent validation include: whether splicing factor concentrations within condensates have been quantitatively measured versus inferred from functional outcomes; whether the proposed dual coupling of transcription and splicing represents a defined spatial architecture or a functional correlation; and whether the observed alternative splicing changes are condensate‐dependent (e.g., through phase separation‐disrupting mutations in SKIP IDRs) rather than reflecting general cold‐induced changes in splicing factor expression. The conceptual parallel to other systems where transcription factors organize post‐transcriptional condensates (cf. FCA/FLL2, Section 4.1.4) is suggestive but requires direct comparative analysis.

### 4.2 Cytoplasmic Condensates

#### 4.2.1 ALBA4, ALBA5, ALBA6 Proteins as Thermosensors

The primary scaffold(s) driving the nucleation of SGs in Arabidopsis heat stress remain incompletely defined, though RBGD2/4 (Section 4.2.2) and OLIGOURIDYLATE‐BINDING PROTEIN 1C (UBP1C, Section 7.3) have been implicated in related systems. ALBA4, ALBA5, and ALBA6 are highly conserved proteins with N‐terminal ALBA domains and C‐terminal RGG motifs [125]. Upon heat stress, ALBA4/5/6 relocalize to SGs and P‐bodies [29]. Yet, ALBA proteins are not required for SG assembly (see below), establishing them as clients rather than scaffolds. Genetic ablation (*alba456* triple mutant) does not prevent SG formation, confirming client status, but confers hypersensitivity to heat stress, demonstrating that client recruitment has direct functional consequences for thermotolerance [29]. Enhanced CrossLinking and ImmunoPrecipitation (eCLIP) analysis of ALBA5 in vivo binding targets revealed enrichment for heat stress, heat acclimation, and oxidative stress transcripts. RNA immunoprecipitation on isolated SGs confirmed ALBA5 binding to HEAT SHOCK FACTOR (HSF) mRNAs within condensates [29]. In *alba456* mutants, HSF mRNA abundance in SGs is reduced but not eliminated, indicating partial redundancy with other SG‐associated RNA‐binding proteins. Critically, heat stress‐related transcripts show shortened half‐lives in *alba456*, and elimination of the cytoplasmic EXORIBONUCLEASE 4 (XRN4) rescues *alba456* heat hypersensitivity [29]. This epistasis establishes a clear mechanistic chain: ALBA‐mediated sequestration of HSF mRNAs within SGs physically protects them from XRN4‐dependent degradation in P‐bodies, directly linking condensate composition to transcript stability and stress survival. This study represents one of the most complete functional demonstrations of protective RNA sequestration within plant condensates.

ALBA proteins also interact with m^6^A reader proteins in Arabidopsis [34], potentially connecting SG‐mediated mRNA protection to epitranscriptomic regulation through ECT condensates (Section 4.2.4). Whether m^6^A modification of HSF transcripts influences their ALBA‐dependent recruitment to SGs remains untested. Furthermore, whether metabolites promote ALBA functions, remains unclear.

#### 4.2.2 RBGD2 and RBGD4 in Heat Stress

RNA‐BINDING GLYCINE‐RICH DOMAIN proteins RBGD2 and RBGD4 are upregulated by heat stress and undergo heat‐induced LLPS in vitro [30]. Domain mapping identified the two N‐terminal RNA recognition motifs (RRMs) and the C‐terminal low‐complexity domain (LCD) as jointly sufficient for phase separation. The IDRs of RBGD2 and RBGD4 contain 22 and 25 tyrosine residues, respectively, consistent with aromatic‐driven π–π stacking as the primary condensation mechanism (cf. Section 3.2). In vivo, RBGD2/4 form cytoplasmic granules within 30 minutes of heat stress onset. Given their in vitro LLPS capacity and rapid granule formation kinetics, RBGD2/4 represent strong candidates for SG scaffolding functions in Arabidopsis, though direct co‐localization analysis has not been reported.

Approximately 68% of RBGD2/4‐associated transcripts are shared between ambient and heat stress conditions, but the heat‐specific fraction is enriched for stress‐responsive mRNAs including *HSFA2* and *HSP70*, indicating active remodeling of the RNA clients upon stress [30]. RBGD2/4 protect these transcripts from degradation, though whether the mechanism involves XRN4 exclusion, as demonstrated for ALBA proteins, remains untested. The protein interactome of RBGD2/4 also remodels upon heat stress, suggesting that both RNA and protein composition of RBGD2/4‐containing granules are dynamically tuned to stress conditions. Furthermore, whether metabolites promote RBGD2/4 functions, remains unclear.

#### 4.2.3 The RNA Chaperone GRP7 in Temperature Sensing

GRP7 contains an N‐terminal RRM and a C‐terminal glycine‐rich IDR sufficient for LLPS both in vitro and in vivo [31, 32, 33]. Tyrosine residues within this G‐rich IDR drive phase separation through aromatic interactions, paralleling RBGD2/4 (Section 4.2.2) but within a distinct sequence context, G‐rich spacers versus LCD spacers, potentially conferring different material properties and client specificities consistent with sticker‐and‐spacer predictions (Section 3). Critically, condensation is gated by phosphorylation: the receptor‐like kinase FERONIA (FER) phosphorylates Ser^132^ and Ser^139^ within the IDR, and this phosphorylation is essential for condensate formation both in vitro and in vivo [31, 32]. GRP7 thus exemplifies how a membrane‐localized kinase can control cytoplasmic condensate assembly, providing a direct mechanistic link between plasma membrane sensing and condensate‐mediated responses, a principle relevant to the 2D to 3D membrane nucleation concept discussed in the Perspective.

Arabidopsis ecotypes Blh‐1 and Bre‐1, native to habitats with smaller daily temperature fluctuations, carry natural polymorphisms at Ser^132^, the FER phosphorylation site [32]. These variants show altered LLPS capacity and fail to complement the *grp7* mutant root growth defects under fluctuating temperatures. Combined with ELF3 polyQ length variation across accessions (Section 4.1.3), this establishes a recurring principle: natural sequence variation at phase separation‐determining residues directly modulates ecological thermal adaptation in plants. Loss‐of‐function *fer* and *grp7* mutants both show attenuated root growth under temperature shifts, confirming the physiological importance of the FER–GRP7 condensation axis [31, 32].

GRP7 condensates recruit translation initiation factor eIF4E1 and RNA chaperones COLD SHOCK PROTEINs 1 and 3 (CSP1/CSP3), creating microenvironments where translation is inhibited during heat stress [32]. GRP7 binds broadly across transcript regions with 3’ untranslated region (UTR) preference [126], and the granules sequester nascent mRNAs. GRP7 additionally regulates alternative splicing in the nucleus through FER‐dependent phosphorylation [31, 32]; whether this nuclear function involves condensate formation or operates through a distinct, non‐phase‐separated mechanism has not been resolved. Specific metabolite enrichment within GRP7 granules has not been characterized.

#### 4.2.4 The m^6^A Readers ECTs as Temperature Sensors

Among the thirteen YT521‐B homology (YTH)‐domain proteins in Arabidopsis, most of which have been confirmed or inferred to bind m^6^A, ECT9 and ECT1 have been directly characterized for condensate formation. ECT9 autonomously forms liquid‐like condensates in vitro and in vivo, requiring cooperation between its N‐terminal IDR and C‐terminal YTH domain, neither domain alone is sufficient [35]. ECT1 presents a more complex picture: Wang et al. (2023a) reported that ECT1 cannot independently form condensates and is recruited into ECT9 condensates through direct protein–protein interactions, whereas Lee et al. (2024) demonstrated that ECT1 undergoes LLPS in vitro and is recruited to P‐bodies and SGs in vivo upon salicylic acid treatment, with its N‐terminal PrLD required for condensation [36]. These findings are not necessarily contradictory but suggest that ECT1 condensation is context‐dependent, negligible under basal conditions yet triggered by immune signaling or molecular crowding. The ECT9–ECT1 relationship may therefore represent a conditional scaffold–client interaction rather than an absolute one, in which ECT9 lowers the condensation threshold for ECT1 under non‐stress conditions, while stress signals independently promote ECT1 phase separation. This context‐dependence aligns with the broader finding that ECT1, ECT9, and ECT11 have undergone lineage‐specific neo‐functionalization, driven primarily by divergent IDR properties, despite retaining m^6^A‐binding capacity [127]. Both proteins contain N‐terminal IDRs enriched in G, S, and R residues; the YTH domain's conserved aromatic cage provides m^6^A‐binding specificity [128, 129], mechanistically decoupling target selection from the condensation process itself.

Temperature stress alters the m^6^A epitranscriptomic landscape, and ECT proteins have been implicated in coordinating adaptive responses [130, 131]. However, several key claims require evidential qualification: whether ECT1/ECT9 physically redistribute to newly m^6^A ‐marked transcripts upon heat stress has not been demonstrated by direct binding assays under stress conditions; whether ECT condensate material properties change with temperature in a manner attributable to disruption of aromatic interactions remains inferential, based on sequence composition rather than biophysical measurement; and whether cold‐induced ECT9 condensates specifically protect cold‐responsive translation [28, 132] requires direct demonstration beyond correlative expression data.

ECT condensates are distinctive in that RNA serves as both a regulatory target and the recruitment signal: the YTH domain recognizes m^6^A‐modified transcripts, making condensate RNA composition dependent on upstream writer activity. This creates a functional link to CRY2/MTA/FIO1 writer condensates (Section 4.1.2), which deposit m^6^A marks on target transcripts under light stimulation, and to ALBA proteins (Section 4.2.1), which interact with ECT proteins via a conserved YAIM motif in the IDR and co‐protect heat stress transcripts [127]. Together, these systems suggest a three‐step condensate cascade: writer condensates (CRY2/MTA) establish epitranscriptomic mark (m^6^A) which, in turn, induces the condensation of readers (ECT9/ECT1). If confirmed as an integrated pathway, this would represent the most complete condensate‐to‐condensate signaling chain characterized in plants; however, no study has yet demonstrated that specific transcripts methylated by the CRY2/MTA writer condensates are subsequently recognized and sequestered by ECT reader condensates, and the cascade remains assembled from independent observations across different experimental systems and conditions. Specific metabolite enrichment within ECT condensates has not been characterized.

### 4.3 Prokaryotic Type of Condensates in Temperature Sensing

#### 4.3.1 The Chloroplastic SCO1

SCO1, a nuclear‐encoded chloroplast elongation factor G, forms heat‐induced foci (chloroplastic SGs, cpSGs) within chloroplasts. cpSGs are detectable from 33°C but were primarily characterized at 42°C (30 min, darkness) [37]. Whether SCO1 functions as the cpSG scaffold or as an abundant client remains unresolved: no in vitro LLPS reconstitution or genetic depletion experiments have been reported. The proteomic analysis reveals multiple IDR‐containing RNA‐binding proteins, the chaperone HSP90‐5, the translation factor RABE1b, and the DEAD‐box helicase RH3 within cpSGs, any of which could contribute to scaffold function [37]. Recruitment of RuBisCO activase and RuBisCO accumulation factors suggests that cpSGs protect and coordinate the photosynthetic machinery during thermal stress, though direct functional evidence (e.g., RuBisCO activity measurements in cpSG‐deficient backgrounds) is lacking.

Specific RNA components of cpSGs have not been individually identified, though RNA‐seq of cpSG isolates detected associated transcripts, and the enrichment of RNA‐binding proteins and a DEAD‐box helicase implies RNA involvement. Whether cpSGs sequester chloroplast‐encoded mRNAs (predominantly polycistronic, intron‐poor transcripts processed by prokaryotic‐type machinery) or nuclear‐encoded mRNAs imported into chloroplasts has not been determined, a distinction with implications for the mechanism of translational protection.

cpSGs are one of two condensate systems in this review with direct metabolomic profiling data, alongside cytosolic SGs [38]. However, the metabolite profiles differ: cytosolic SGs contain nucleotides (including adenine dinucleotide phosphate), amino acids (proline, glutamic acid, leucine, methionine), and phospholipid precursors (citicoline, phosphoethanolamine) [38], whereas cpSGs contain fatty acids (stearic acid, palmitic acid) and two amino acids (glutamic acid, proline) [37]. The shared enrichment of proline and glutamic acid across both compartments is notable: proline has documented roles as a chemical chaperone and osmolyte, though whether proline concentrations within cpSGs exceed stromal baseline levels, as opposed to reflecting passive co‐purification, requires quantitative comparison. The fatty acid enrichment in cpSGs, distinct from the phospholipid precursor signature of cytosolic SGs, is notable but requires cautious interpretation: palmitic and stearic acids are among the most abundant metabolites in the chloroplast stroma as substrates for de novo fatty acid biosynthesis and thylakoid lipid assembly, and whether their cpSG enrichment reflects specific retention versus co‐purification of stroma or membrane‐derived material remains unresolved.

Phase separation within chloroplasts operates under physicochemical conditions distinct from the cytoplasm: high stromal Mg^2+^ concentrations, pH gradients across thylakoid membranes, and extremely high local protein concentrations (RuBisCO alone reaches ∼500 µm in the stroma). How these parameters influence *C*_sat_ for cpSG scaffolds is unknown. Notably, cpSGs (heat‐induced, SCO1) and CP29A condensates (cold‐induced; Section 4.3.2) represent LCST and UCST behavior, respectively within the same organelle, demonstrating that chloroplasts employ both thermodynamic regimes for temperature‐responsive phase separation.

cpSG characterization derives from a single study [37]. The system currently lacks in vitro reconstitution, fluorescence recovery after photobleaching (FRAP) measurements, and fusion/fission dynamics data, criteria that would be required to classify cpSGs as bona fide LLPS condensates rather than heat‐induced aggregates or protein assemblies formed through alternative mechanisms (cf. field standards discussion, Perspective). The ‘SG‐like’ designation [37] appropriately reflects this evidential status.

#### 4.3.2 The Chloroplastic CP29A

The CP29A is a chloroplast‐localized RNA‐binding protein with a modular architecture: a PrLD sandwiched between two RRM domains [39]. The PrLD mediates LLPS at low temperatures consistent with UCST behavior, demonstrated both in vitro and in vivo under cold stress [39]. Nuclear magnetic resonance (NMR) analysis revealed that the PrLD maintains conformational dynamics at low temperatures while charged and polar residues form transient electrostatic networks, interactions that are enthalpically strengthened at reduced thermal energy, driving condensate assembly through the UCST mechanism [39]. However, whether the NMR‐observed conformational behavior reflects bona fide LLPS with a defined phase boundary, or local structuring and clustering below the demixing threshold, has not been conclusively resolved. This contrasts with SCO1 cpSGs (Section 4.3.1), which form under heat stress through an apparently LCST‐type mechanism, together, CP29A and SCO1 demonstrate that chloroplasts employ both thermodynamic regimes for temperature‐responsive condensation within a single organellar compartment.

Specific protein clients recruited into CP29A condensates have not been individually identified, though the concentration of RNA processing machinery adjacent to nucleoids implies recruitment of processing and maturation factors. Identification of these components, under cold stress, would clarify the client composition.

The dual RRM domains provide sequence‐specific binding with preference for polyU motifs, and CP29A condensates recruit predominantly intron‐less chloroplast transcripts [39]. CP29A binds preferentially to the 5’‐UTR of *RBCL* mRNA, supporting RuBisCO large subunit expression and accumulation during cold acclimation [40]. *cp29a* mutants show impaired splicing of intron‐containing chloroplast genes and global translational reduction, causing cold sensitivity and defective chloroplast biogenesis [39, 133]. However, since most CP29A RNA targets are intron‐less, the splicing defect likely reflects an indirect consequence of disrupted chloroplast gene expression rather than a direct condensate‐mediated splicing function. The condensate‐specific RNA functions, stability maintenance, translational timing, and degradation protection for intron‐less transcripts, are inferred from binding data and mutant phenotypes but have not been demonstrated through condensate‐disrupting mutations that preserve CP29A's RNA‐binding activity.

Specific metabolite enrichment within CP29A condensates has not been characterized. Given that SCO1 cpSGs show amino acid and nucleotide enrichment (Section 4.3.1), metabolomic profiling of CP29A condensates would determine whether chloroplastic condensates share a similar metabolite signature.

#### 4.3.3 Chloroplast Protein Sorting With STTs

The ankyrin‐repeat proteins SORTING TO THYLAKOIDS 1 (STT1) and STT2 form heterodimeric complexes through their C‐terminal IDRs and undergo a putative substrate‐induced LLPS, a distinctive trigger mechanism where client binding itself drives phase separation rather than environmental conditions [41]. The STT1 IDR contributes a negatively charged WEEPD motif that engages positively charged twin‐arginine (RR) residues in substrate lumen targeting peptides, while the STT2 IDR provides a hydrophobic LVP‐W motif that interacts with substrate hydrophobic cores [41]. This dual electrostatic‐hydrophobic recognition enables 1:1 stoichiometric binding that triggers condensate formation only when substrates are available, an elegant substrate‐gated condensation mechanism.

Validated chloroplast twin‐arginine translocation (cpTat) pathway substrates recruited into STT condensates include oxygen‐evolving complex subunits OE23, OE17, and photosystem I subunit PsaN [41]. The condensate concentrates these substrates for delivery to the thylakoid membrane cpTat translocon. Crucially, binding to the translocon receptor component Hcf106 dissolves the condensate and releases substrates for translocation [41]. This target‐triggered dissolution is mechanistically distinct from the environment‐driven dissolution seen in most other systems in this review (e.g., thermal reversion dissolving PhyB photobodies, warming dissolving GI condensates): here, condensate lifetime is determined by delivery kinetics rather than by reversal of assembly conditions. Genetic evidence confirms functional importance: *stt1* and *stt2* knockdown lines show selectively compromised cpTat substrate transport, defective chloroplast biogenesis, and cold sensitivity [41, 42].

No RNA component has been identified for this system. STT condensates appear to operate as a purely protein‐based phase separation system, unusual among the condensates reviewed here, most of which involve RNA as either a structural component or regulatory target. Furthermore, the cpTat pathway is driven by the thylakoid proton motive force (PMF), coupling transport activity to photosynthetic light reactions [134]. Whether PMF fluctuations directly modulate STT condensate dynamics, as opposed to acting downstream at the translocon dissolution step, has not been experimentally distinguished. Similarly, while stromal ATP/ADP ratios, ion concentrations (Mg^2+^, K^+^), and redox state fluctuate during environmental transitions and could in principle influence STT phase behavior through effects on IDR electrostatics, direct measurements of STT condensate properties as a function of these parameters have not been reported. The cold sensitivity of *stt* mutants likely reflects compounded effects of reduced PMF and increased demand for photosystem repair substrates, but whether cold directly impairs STT condensate formation or only downstream translocation requires separation.

## 5 Redox Regulation and Nuclear Gating

ROS modulate condensate assembly through direct chemical modification of phase separation‐determining residues, creating a rapid and reversible signaling layer that couples cellular redox state to condensate dynamics [14, 43]. In the systems described below, three distinct redox mechanisms operate. Cysteine oxidation drives condensation: TMF contains four IDR‐localized cysteines that form H_2_O_2_‐induced intermolecular disulfide bonds, concatenating TMF molecules to elevate effective IDR concentration above *C*_sat_ and triggering nuclear condensate assembly (Section 5.1) [43]. The NPC constitutes the obligate gateway through which redox‐regulated cargoes (Section 5.2), including NPR1 monomers released by Cys^82^/Cys^216^ reduction (Table 1) and LSD1 complexes directed by importin α under reducing conditions (Section 5.3), access the nucleus; its selective barrier is itself a phase‐separated assembly whose properties are environmentally modulated. Cysteine redox state gates compartmentalization: the LSD1/CAT2/ PEROXIN5 (PEX5) ternary complex distributes between nucleus, peroxisomes, and cytosol as a function of progressive oxidation, with reducing conditions favoring nuclear import and increasing oxidation sequentially exposing peroxisomal then cytosolic targeting (Section 5.3). Cysteine clusters control oligomeric state: NPR1's Cys^82^ and Cys^216^ regulate the oligomer‐to‐monomer transition that determines nuclear translocation and immune condensate formation (referenced in Table 1) [14]. Beyond proteins, oxidative modifications of RNA bases (8‐oxoguanosine) can alter RNA secondary structure and protein‐binding properties [135, 136], though whether this mechanism operates in any characterized plant condensate system remains undemonstrated. The reversibility of disulfide bonds through cellular reducing systems (glutathione, thioredoxin) enables rapid condensate dissolution during stress recovery [137, 138], establishing a redox‐dependent assembly/disassembly cycle.

### 5.1 TMF

TMF is a plant‐specific ALOG family transcriptional regulator that controls shoot apical meristem maturation through transcriptional repression of the floral specification gene *ANANTHA* (*AN*) [13, 44, 139, 140]. TMF contains an ALOG DNA‐binding domain and an IDR harboring four cysteine residues (C^112^, C^117^, C^124^, and C^126^) that serve as the redox‐sensitive condensation switch [141]. Under reducing conditions, TMF remains dispersed in the nucleoplasm. Upon H_2_O_2_ accumulation, particularly in boundary domains of the shoot apical meristem, these cysteines form intermolecular disulfide bonds that concatenate TMF molecules, elevating the effective local IDR concentration above *C*_sat_ and driving LLPS into nuclear condensates [13, 44, 139, 140]. This mechanism is reversible: reduction of disulfide bonds by cellular reducing systems dissolves condensates, establishing a redox‐dependent assembly/disassembly cycle directly coupled to ROS dynamics during meristem maturation.

TMF recruits transcriptional cofactors including BLADE‐ON‐PETIOLE (BOP) family proteins and TMF‐family paralogs (TFAM1–TFAM3, defined in [141]) homologs into heterotypic condensates through ALOG domain‐mediated protein–protein interactions [141, 142]. BOP incorporation into condensates per se (as opposed to TMF–BOP interaction independent of phase separation) has not been directly demonstrated. Within condensates, TMF binds the *AN* promoter, concentrating transcriptional repression machinery at this locus. The condensate environment enhances TMF's DNA‐binding capacity and promoter dwell time, amplifying repression beyond what dispersed TMF achieves.

Neither RNA components nor metabolite enrichment have been identified within TMF condensates. Current evidence supports a DNA‐binding transcriptional mechanism rather than RNA client recruitment or processing, making TMF condensates an incomplete case within the scaffold–client–RNA–metabolite framework, with only the scaffold and protein client layers experimentally characterized.

TMF is one of several condensate systems operating in meristematic niches: SFH8 undergoes separase‐mediated liquid‐to‐solid transitions at the plasma membrane to establish PIN2 polarity in root meristems [50], and ARF7/ARF19 form cytoplasmic condensates sequestering auxin‐responsive transcription factors (Section 3). Whether these meristematic condensate systems respond to shared environmental inputs (e.g., ROS gradients that regulate both TMF condensation and root meristem patterning) or operate independently remains an open question.

### 5.2 NPC: FG‐Nup Hydrogel as a Phase‐Separated Selective Barrier

The NPC central channel is formed by FG‐nucleoporins (FG‐Nups), particularly the nucleoporin NUP62 subcomplex (NUP54, NUP58, NUP62), whose intrinsically disordered FG‐repeat domains undergo LLPS to form a hydrogel‐like selective barrier [45]. This represents a percolation‐type assembly (cf. Section 3.1) rather than classical liquid droplet formation: FG‐repeat aromatic interactions (π–π stacking between phenylalanine residues) create a system‐spanning network that functions as a molecular sieve, freely permeable to small molecules but requiring nuclear transport receptors (NTRs) for passage of larger macromolecules. The F‐to‐S mutation in NUP62 (Nup62FmuS) abolishes phase separation and barrier function, directly demonstrating that aromatic‐driven percolation is essential for NPC selectivity [45, 143, 144].

Within the condensate framework, NTRs (karyopherin‐β family) function as transient clients that dissolve into the FG‐hydrogel through multivalent weak interactions to escort cargo. Critically, cargo molecules transiting the NPC (e.g., transcription factors, mRNA–protein complexes) interact with the hydrogel barrier but are not condensate components, a distinction between condensate clients (which partition into the phase) and transport cargo (which traverse it). mRNA export through the NPC is a transport function of the hydrogel barrier, not an RNA condensate component. No specific metabolite enrichment within the FG‐hydrogel has been reported, though the energy landscape of NTR–FG interactions depends on the nucleotide state of the Ran GTPase cycle that maintains transport directionality.

NPC composition is not static. At elevated temperatures (28°C vs. 23°C), outer ring nucleoporins NUP85 and NUP133 become specifically required for mRNA export, revealing temperature‐dependent remodeling of NPC function [145]. Whether this reflects altered phase properties of the FG‐hydrogel itself or changes in outer ring scaffold interactions remains unresolved. A mechanistically distinct phenomenon at the NPC involves the KARYOPHERIN 120/MODIFIER of SNC1 4 (KA120/MOS4), which prevents condensation of the MOS4‐associated complex (MAC), a splicing regulatory complex, in the nucleoplasm [146]. This represents a rare, documented example of a protein actively suppressing phase separation of a partner complex, maintaining MAC in a dispersed, functional state. This is the inverse of the scaffold‐driven condensation logic that dominates this review.

### 5.3 CAT2 and LSD1

LSD1 functions as the scaffold, recruiting CAT2 and the peroxisomal import receptor PEX5 as clients into a ternary condensate with documented compositional layering: LSD1 occupies the core, PEX5 coats the surface, and CAT2 binds peripherally through PEX5 [46]. This proposed compositional layering based on differential accessibility (core‐shell architecture) is unique among plant condensates described and implies that client accessibility is spatially regulated within the condensate; PEX5's surface localization positions it for recognition by import receptors, while LSD1's core position may protect it from premature modification.

The LSD1/CAT2 system converts a continuous cellular redox gradient into three discrete compartmental states [46]. Under reducing conditions, LSD1 binds importin α, directing the complex to the nucleus. Under moderate oxidation, PEX5 becomes more accessible on the condensate surface, redirecting import toward peroxisomes and enhancing CAT2 catalase activity at its functional site. At high oxidation levels, peroxisomal import is blocked entirely, retaining the complex in the cytosol. This three‐state switching operates through progressive exposure of different targeting signals as a function of oxidation level, mechanistically analogous to the *C*_sat_ threshold concept (Section 3) but operating through redox chemistry rather than concentration: each compartmental transition requires crossing a distinct redox threshold, effectively creating a multi‐level nonlinear filter that reads oxidative stress intensity and deploys antioxidant defense to the compartment where it is most needed. Note that LSD1 and CAT2 do not directly interact, as PEX5 is the obligate bridge in the ternary complex.

No RNA component has been identified for this system. H_2_O_2_ (a metabolite) is both the environmental signal driving condensate redistribution and the enzymatic substrate of the CAT2 client, a dual role that raises the question of whether H_2_O_2_ is locally concentrated or excluded within the condensate phase, with direct implications for catalytic efficiency. In vitro, LSD1 co‐assembly significantly enhances CAT2 activity [46], though the mechanism of enhancement is unclear; whether is through substrate concentration, conformational stabilization, or protection from inhibitors, has not been determined.

The layered architecture, three‐state redox switching, and activity enhancement derive from a single study [46]. Independent validation, particularly of the in vivo redox‐dependent compartmental switching and of the layered condensate organization by super‐resolution microscopy, would strengthen these conclusions.

## 6 Membrane Association of Condensates as Environmental Sensor

Biomolecular condensates increasingly blur the boundary between membrane‐bound and membrane‐less compartments: multiple plant condensate systems form at or interact with cellular membranes through surface wetting, lipid domain co‐organization, and protein‐lipid binding (reviewed in [147]). In plant cells, the cell wall–plasma membrane interface is the primary site of environmental perception, where receptor‐like kinases, mechanosensitive channels, and lipid‐modifying enzymes transduce external signals. Whether membrane‐associated condensates directly participate in this sensory function, converting mechanical, osmotic, or chemical signals at the membrane into cytoplasmic phase separation responses, remains one of the most important open questions in plant condensate biology.

Several plant membrane‐condensate systems have been identified, though their environmental responsiveness is incompletely characterized. TPLATE condensates at clathrin‐coated pits couple phase separation to endocytic membrane curvature [148, 149]. The VAMP‐Associated Protein 27 (VAP27) ‐ Suppressor of cAMP Receptor/Wiskott–Aldrich syndrome protein family verprolin‐homologous (SCAR/WAVE) system demonstrates endoplasmic reticulum–plasma membrane (ER‐PM) contact site nucleation of condensates (e.g., of P‐bodies through DCP1 [48, 150], with a putative positive feedback between membrane architecture and condensate assembly [48, 151, 152]. Rapid Alkalinization Factor (RALF)‐pectin interactions at the cell wall–plasma membrane junction recruit FERONIA receptor complexes [2]. Below, we examine three membrane‐associated condensate systems in detail, the endomembrane ADAPTOR PROTEIN COMPLEX 3 β‐subunit (AP‐3β) system (Section 6.1), the ENDOSOMAL FYVE DOMAIN PROTEIN REQUIRED FOR ENDOSOMAL SORTING 1 (FREE1) condensate (Section 6.2), and briefly consider RNA‐membrane interactions (Section 6.3). For the Feronia/GRP7 system, where membrane‐localized kinase activity directly gates cytoplasmic condensate formation, see Section 4.2.3.

### 6.1 Endomembrane System and AP‐3β

The AP‐3β, canonically a component of endomembrane trafficking machinery at the *trans*‐Golgi network [153], exhibits an unconventional function in condensate dynamics: upon heat stress, AP‐3β rapidly relocalizes to SGs, where it interacts with the RNA‐binding proteins TUDOR STAPHYLOCOCCAL NUCLEASE  1/2 (TSN1/TSN2) and facilitates SG disassembly during stress recovery [47]. Mechanistically, AP‐3β recruits the 19S regulatory particle of the proteasome to SGs, promoting deubiquitylation of SG components and facilitating SG clearance required for translation resumption. AP‐3β thus functions not as a scaffold or client within the assembly framework but as a disassembly factor, a functional category that the scaffold–client–RNA–metabolite framework does not capture, highlighting that condensate regulation requires both assembly and disassembly machinery.

This finding completes a SGs lifecycle model when combined with other systems in this review: RBGD2/4 provide scaffold‐driven SG nucleation (Section 4.2.2), ALBA4/5/6 mediate mRNA protection within assembled SGs (Section 4.2.1), and AP‐3β/19S drives SG dissolution during recovery. AP‐3β uses its C‐terminal domain for TSN1/2 interaction, but membrane association properties, involving the IDR region, contribute to SG recruitment.

No RNA or metabolite enrichment specific to AP‐3β’s SG disassembly function has been characterized. Whether AP‐3β’s recruitment to SGs is triggered directly by temperature recovery or indirectly through restoration of proteostatic capacity is not known, a distinction that would clarify whether this represents environmental sensing or a downstream housekeeping function.

### 6.2 FREE1

FREE1 is a plant‐specific endosomal sorting complex required for transport (ESCRT) component whose IDR drives LLPS at endosomal membranes [49, 154]. FREE1 binds phosphatidylinositol‐3‐phosphate (PI3P)‐enriched membranes via its FYVE domain, positioning the IDR‐driven condensate at the multivesicular body (MVB) surface. The condensate‐membrane interaction generates capillary forces that drive membrane invagination, followed by wetting‐induced line tension forces sufficient to mediate neck scission [49]. This makes FREE1 the only system in this review where phase separation directly performs a mechanical membrane function, converting thermodynamically driven assembly into physical membrane deformation. Plants expressing FREE1 lacking the IDR show hypersensitivity to hyperosmotic stress, linking condensate‐mediated scission to hormone homeostasis through impaired multivesicular bodies (MVBs)‐dependent degradation of abscisic acid (ABA) signaling components [49].

FREE1 associates with the ESCRT‐I complex through direct interaction with vacuolar protein sorting 23 (VPS23), initiating incorporation of ubiquitinated cargo proteins (PIN‐FORMED 2 (PIN2), IRON‐REGULATED TRANSPORTER 1 (IRT1), PYRABACTIN RESISTANCE/PYRABACTIN‐LIKE ABA receptors (PYR/PYL)) into the sorting pathway [155, 156, 157]. Whether these cargo proteins partition into the FREE1 condensate phase or are processed at the condensate‐membrane interface without entering the phase‐separated domain has not been distinguished, a question with implications for whether the condensate functions as a concentrating platform or a mechanical actuator. Furthermore, FREE1 plays additional roles in autophagosome closure, iron starvation responses, and protein homeostasis [154, 155, 156, 157], though whether these functions require FREE1's phase separation activity or operate through its canonical ESCRT protein–protein interactions has not been resolved.

No RNA component has been identified within FREE1 condensates at MVBs. However, proteomic analysis of P‐bodies revealed enrichment of FREE1 and other ESCRT components [150, 158], raising the possibility that FREE1 partitions into RNA‐containing condensates in addition to its membrane‐associated function. Whether FREE1 uses the same IDR for both MVB‐associated LLPS and P‐body partitioning, and whether its condensate function is active in P‐bodies, remains untested [159].

PI3P functions as a membrane‐localized metabolite that targets FREE1 condensation to specific endosomal compartments via FYVE domain binding. This lipid‐dependent targeting represents a distinct mechanism from the soluble‐phase condensation seen in most systems in this review: the metabolite does not enrich within the condensate but rather determines where condensation occurs.

### 6.3 RNA‐Membrane Interactions

Whether RNA can directly scaffold condensates at membranes, independent of protein‐mediated recruitment, remains an open question in condensate biology. In vitro studies have demonstrated direct RNA‐lipid interactions dependent on nucleotide content and sequence composition [160], but the concentrations required substantially exceed physiological conditions, and no plant system has been identified where RNA‐membrane interactions drive condensate formation independently of RNA‐binding proteins. In the plant membrane‐associated systems described above, RNA involvement is either absent (STT1/STT2), uncharacterized (FERONIA‐associated condensates), or mediated entirely through protein intermediaries (P‐bodies). Identifying whether plant membrane‐associated condensates contain specific RNA components, and whether those RNAs contribute structurally to condensate‐membrane interactions, represents a gap that targeted RNA immunoprecipitation from purified membrane fractions could address.

## 7 Nutrient Stress and Hypoxia

Nutrient limitation and oxygen deprivation challenge plant cells through a partially shared physicochemical axis: energy depletion. Reduced ATP availability, most directly from hypoxia blocking oxidative phosphorylation or phosphate deficiency impairing ATP synthesis, and more indirectly from nitrogen starvation curtailing metabolic capacity, impacts condensate biology through at least three interconnected mechanisms. First, declining adenylate energy charge removes the ATP‐dependent processes (chaperone remodeling by Hsp70/Hsp104 systems, RNA helicase activity, kinase‐mediated dissolution) that actively maintain condensates in dynamic liquid states, favoring maturation toward less reversible gel‐like or solid assemblies ([75]; cf. Section 3). Second, global translation arrest triggered by the energy crisis releases mRNAs and RNA‐binding proteins from polysomes, raising their effective cytoplasmic concentration above the saturation concentration (*C*_sat_) required for stress granule nucleation. Third, stress‐associated changes in intracellular physicochemistry, cytoplasmic acidification during energy depletion, altered ionic strength, amino acid pool rebalancing, shift the solvent conditions that define phase boundaries, as demonstrated by pH‐driven phase separation of Sup35 in nutrient‐starved yeast [104] and thermal/pH‐dependent demixing of Pab1 under physiological stress [161]. In plants, compositional profiling of stress granules has confirmed the presence of nucleotides, amino acids, and phospholipids within condensate interiors [38], consistent with metabolite partitioning into condensates, although direct demonstration of solvent‐property‐driven phase boundary shifts remains lacking in any plant system.

Below, we examine three nutrient stress‐responsive condensate systems: MED19a/ ORESARA1 (ORE1) transcriptional condensates that coordinate nitrogen starvation‐induced senescence through PTM‐gated LLPS (Section 7.1), nutrient stress‐induced stress granules whose composition and dynamics are detailed by proteomics and metabolomics (Section 7.2), and UBP1C‐scaffolded stress granules that mediate selective mRNA triage during hypoxia (Section 7.3).

### 7.1 The Mediator Complex

MED19a contains an MC‐IDR with alternating clusters of positive and negative residues that drive LLPS through electrostatic interactions [51]. Condensation is gated by a PTM switch: under nitrogen‐sufficient conditions, MED19a lysine hyperacetylation neutralizes positive charges within the MC‐IDR, suppressing phase separation. Nitrogen starvation triggers deacetylation, restoring the charge pattern and enabling condensate assembly, directly coupling nitrogen availability to transcriptional condensate formation [51]. The specific acetyltransferases and deacetylases mediating this switch, and whether they respond to nitrogen metabolites directly or through upstream signaling, remain uncharacterized. The MC‐IDR is functionally conserved: human cMED19 MC‐IDR partially complements Arabidopsis *med19a* mutants [51], though whether this complementation requires condensate formation or reflects conserved protein–protein interactions independent of LLPS has not been tested.

The master senescence transcription factor ORESARA1 (ORE1) is recruited into MED19a condensates to form heterotypic transcriptional assemblies at senescence‐associated gene promoters, activating programs for chlorophyll catabolism, programmed cell death, and nitrogen remobilization from source leaves to developing tissues [51]. As a Mediator subunit, MED19a is expected to bridge ORE1 to the core Mediator complex and RNA Pol II; however, direct visualization of additional Mediator subunits or Pol II within MED19a condensates has not been reported.

The root‐derived long non‐coding RNA ELF18‐INDUCED LONG NONCODING RNA 1 (*ELENA1*) is induced under nitrogen deficiency and transported systemically from roots to shoots, where it dissociates the MED19a‐ORE1 complex, calibrating senescence progression to prevent premature leaf death [52]. This represents the only documented case in this review of a systemically mobile RNA regulating a condensate‐associated transcriptional complex from a remote tissue, establishing a root‐to‐shoot communication axis that tunes condensate‐mediated transcription to whole‐plant nitrogen status. Whether *ELENA1* causes dissolution of MED19a condensates or selectively dissociates ORE1 from within intact condensates has not been distinguished. Reciprocal grafting of wild‐type and elena1 mutants confirmed systemic *ELENA1* movement [52], but whether *ELENA1* directly binds MED19a or ORE1, the carrier mechanism mediating phloem transport, and whether *ELENA1* dissociates the MED19a–ORE1 protein interaction or triggers dissolution of the condensate itself, remain unresolved.

Nucleotide substrates (ATP, GTP, CTP, UTP) for RNA Pol II are presumably accessible within transcriptional condensates, but metabolomic profiling has not been performed. The nitrogen metabolites whose depletion triggers MED19a deacetylation, whether glutamine, nitrate, or other nitrogen‐status indicators, are unknown, leaving a gap between the environmental signal (nitrogen limitation) and the PTM response (deacetylation).

### 7.2 SGs in Nutrient Stress

TSN1/TSN2 serve as docking platforms for SG assembly, with their tandem SN domains, which are highly intrinsically disordered, mediating protein–protein interactions and SG localization, and their Tudor domains predicted to recognize methylated arginine on RNA‐binding proteins based on structural conservation with mammalian orthologues [53, 54, 162]. The SN domains additionally contribute to mRNA stability regulation within the condensate [163, 164]. TSN scaffolds recruit SNF1‐RELATED KINASE 1 (SnRK1), the plant orthologue of AMP‐ACTIVATED PROTEIN KINASE (AMPK), directly linking SG formation to the central energy‐sensing kinase network: both TSN and SG formation are required for heat‐induced SnRK1 activation [54]. Whether this TSN‐dependent SnRK1 activation also operates under nutrient stress, and whether SnRK1 is activated within SGs, for instance by sensing local metabolite concentrations, or is sequestered there to attenuate its cytoplasmic activity, has not been experimentally distinguished. The relationship between TSN‐nucleated nutrient SGs and the RBGD2/4‐scaffolded heat SGs (Section 4.2.2) is unclear: whether these represent the same condensate type assembled under different stresses, or structurally distinct granules with shared clients, remains an important unresolved question.

Proteomic analysis reveals recruitment of translation initiation factors (eIF3, eIF4E, eIF4G, 40S subunits), poly(A)‐binding proteins (PABP2/4/5/8), CCCH‐zinc finger proteins including TANDEM ZINC FINGER 1 (TZF1) that shuttle between nucleus and SGs/P‐bodies [163], DEAD‐BOX RNA HELICASES (RH6/RH8/RH12), and metabolic enzymes including glutamine synthetase [13, 38, 53, 54, 162, 165]. The breadth of this client network, spanning translation, RNA processing, and metabolism, suggests nutrient SGs function as multi‐pathway coordination hubs rather than simple translational storage sites.

SGs selectively sequester mRNAs based on *cis*‐acting elements including U‐rich 3’ UTR motifs, AU‐rich elements, and structural features in untranslated regions [53, 162]. In yeast and mammalian SGs, highly induced stress‐responsive transcripts largely circumvent sequestration and remain polysome‐associated, while constitutive housekeeping mRNAs are preferentially stored, an energy‐saving triage mechanism [166, 167]. Whether this selective triage operates similarly in plant nutrient SGs has not been directly tested. The DEAD‐box helicases RH6/8/12 associate with both PBs and SGs and regulate the turnover of stress‐responsive mRNAs [168], though their specific role in controlling mRNA exchange dynamics within nutrient SGs has not been directly demonstrated. Sequestered mRNAs undergo one of three fates: translational repression with storage, degradation via P‐body‐associated XRN4, or protection through association with stabilizing RNA‐binding proteins. Upon stress relief, mRNA recovery for translation has been documented in yeast and mammalian systems, and the presence of intact mRNAs together with translation initiation factors within plant SGs [38] is consistent with a primarily reversible storage function, though direct translational recovery kinetics have not been measured in plant nutrient SGs.

Nutrient SGs are one of only two condensate systems in this review (alongside SCO1 cpSGs, Section 4.3.1) with direct metabolomic profiling data. Affinity purification coupled with metabolomics revealed enrichment of nucleotides (ATP, GTP, ADP, AMP, NAD^+^), multiple proteinogenic amino acids, and phospholipids [38]. The signaling metabolite 2’,3’‐cyclic AMP promotes SG assembly by binding the SG scaffold Rbp47b and enhancing its self‐oligomerization, functioning as a small‐molecule cofactor that augments multivalent interactions driving phase separation [9, 37, 38, 90, 169]. Separately, in yeast, S‐adenosylmethionine (SAM) is a conserved metabolite regulator of SG assembly composition [91], raising the question of whether analogous methylation‐dependent mechanisms operate in plant SGs. ATP serves dual roles: maintaining liquid‐like condensate dynamics through DEAD‐box helicase activity and potentially buffering local energy supply against global ATP depletion during nutrient stress. Whether the metabolite enrichment reflects passive concentration through macromolecular crowding or active retention through enzyme–substrate binding, and whether glutamine synthetase sequestration creates functionally significant metabolic microenvironments, requires quantitative comparison of intra‐condensate versus cytoplasmic metabolite concentrations.

### 7.3 UBP1C and Hypoxia

UBP1C contains three RRMs functionally analogous to mammalian TIA‐1/TIAR and functions as the primary scaffold for SG assembly during hypoxia [55, 168, 170, 171]. Under normoxic conditions, UBP1C binds >1000 mRNAs with U‐rich 3’ UTRs in a dispersed nucleocytoplasmic distribution. Upon oxygen deprivation, UBP1C undergoes rapid relocalization (within minutes) to cytoplasmic foci, coinciding with polysome disassembly and requiring ribosome run‐off [55]. Formation is triggered broadly by energy crisis rather than hypoxia specifically: oxidative phosphorylation inhibitors (arsenite, cyanide, myxothiazol) all induce UBP1C foci, and granules disassemble rapidly (within minutes) upon reoxygenation [55].

UBP1C SGs share extensive client overlap with the nutrient SGs described in Section 7.2, including translation initiation factors, PABPs, and RH6/8/12 RNA helicases [90, 172, 173], suggesting a common SG architecture recruited under diverse energy‐depleting conditions. Hypoxia‐specific clients include UBP1‐associated proteins UBA1a/UBA2a and the Ca^2+^ sensor CML38 [5]. CML38's presence is notable as one of the few documented Ca^2+^‐condensate connections in plants; whether CML38 actively regulates SG dynamics through Ca^2+^‐dependent conformational changes or is passively sequestered has not been tested. Upstream autophagy proteins (ATG1a, ATG13a, ATG6, VPS34, ATG5) translocate to SGs during heat stress and re‐translocate upon recovery to facilitate rapid autophagy activation [174]; whether this priming mechanism also operates in hypoxia SGs remains to be demonstrated.

During hypoxia, UBP1C shifts from sequence‐specific U‐rich 3’ UTR binding to promiscuous association with non‐U‐rich transcripts, implementing the same selective triage logic described for nutrient SGs (Section 7.2): constitutive mRNAs are preferentially sequestered, while highly induced hypoxia‐responsive transcripts such as *ALCOHOL DEHYDROGENASE 1* (*ADH1*) bypass sequestration and remain polysome‐associated [55]. This energy‐saving strategy ensures translation is restricted to essential stress‐response proteins.

Direct metabolomic profiling of UBP1C SGs has not been performed, in contrast to the nutrient SG data described in Section 7.2. The intimate coupling between UBP1C condensation and cellular energy status (ATP depletion, oxidative phosphorylation blockade) strongly predicts ATP‐dependent regulation of condensate dynamics, and 2’,3’‐cAMP interaction with RNA‐BINDING PROTEIN 47B (RBP47B) during SG formation provides indirect evidence for signaling metabolite involvement [169]. Hypoxia triggers extensive metabolic remodeling, accumulation of succinate, alanine, gamma‐aminobutyric acid, and ethanol alongside ATP depletion [175, 176, 177], but whether any of these metabolites directly influence UBP1C phase behavior, or are merely consequences of the same energy crisis that triggers SG formation, has not been distinguished. Definitive characterization will require metabolomics of purified hypoxia SGs or deployment of fluorescent metabolite sensors within condensates.

## 8 Perspective

This review has organized plant condensate systems through a unified scaffold–client–RNA–metabolite framework applied across environmental stimuli. Several cross‐system principles emerge from this comparative analysis that define both the field's achievements and its most pressing gaps.

Condensate specificity remains poorly understood. How condensates achieve selective client recruitment while avoiding promiscuous sequestration is a central unresolved question. The systems reviewed here suggest multiple specificity mechanisms operating at different scales: electrostatic complementarity between scaffold IDRs and client motifs (STT1/STT2 WEEPD–RR recognition, Section 4.3.3), hierarchical assembly where core scaffold oligomerization is separable from RNA‐dependent higher‐order condensation (FCA tetramer to FLL2‐dependent multimers, Section 4.1.4), and epitranscriptomics where upstream writer condensates (CRY2/MTA) determine the m^6^A marks that downstream reader condensates recognize, with ECT9 functioning as the primary scaffold and ECT1 recruited as a context‐dependent client whose independent condensation capacity is stress‐conditional (Sections 4.1.2 and 4.2.4). Dissecting these mechanisms quantitatively, including measurement of client partition coefficients, binding affinities within versus outside condensates, and residence times, will be essential for predictive models of condensate composition.

Natural variation provides direct evidence for adaptive significance. Two independent systems demonstrate that natural sequence variation at phase separation‐determining residues modulates ecological fitness: ELF3 polyQ tract length determines the critical temperature for LCST condensation across Arabidopsis accessions (Section 4.1.3), and GRP7 Ser^132^ polymorphisms in ecotypes from thermally stable habitats alter FERONIA‐dependent LLPS capacity and temperature adaptation (Section 4.2.3). These findings establish that condensate phase boundaries are under natural selection, providing the strongest available evidence that phase separation is not a biophysical curiosity but an ecologically relevant trait. Extending this analysis to crop species, where IDR sequence features could be rationally modified to tune condensate‐mediated stress thresholds for specific agricultural environments, represents a concrete translational opportunity grounded in the physicochemical principles described in this review.

Standardized criteria combining in vitro reconstitution, in vivo biophysical measurements (FRAP, optodroplet assays), and genetic validation through phase separation‐disrupting mutations that preserve other protein functions would strengthen the field. Among the systems reviewed here, MED19a condensates (Section 7.1) rely on fluorescent puncta formation and 1,6‐hexanediol sensitivity without in vitro reconstitution or FRAP; UBP1C granules (Section 7.3) show rapid reversibility consistent with LLPS but lack formal biophysical characterization (fusion, phase diagram); and the STT1/STT2 cpTat assemblies (Section 4.3.3) have not been tested for any LLPS criteria beyond co‐localization. Our evidence class annotations throughout this review (Table 1) systematically flag these and other gaps across all systems. Membrane‐to‐cytoplasm condensate nucleation is an emerging frontier. Two‐dimensional phase separation at plasma membrane nanodomains could lower cytoplasmic *C*_sat_ through receptor clustering that increases local scaffold valency, coupling membrane sensing to cytoplasmic condensate formation. The FERONIA/GRP7 system (Section 4.2.3) exemplifies this principle: a membrane‐localized kinase directly gates cytoplasmic scaffold condensation through phosphorylation. Systematic investigation of how lipid composition, membrane curvature, and receptor density modulate 2D‐to‐3D condensate transitions represents a promising direction for understanding the earliest steps of environmental signal transduction.

Chloroplasts may represent an excellent physicochemical test bed. The co‐existence of LCST‐type (SCO1 cpSGs, heat‐induced) and UCST‐type (CP29A, cold‐induced) condensates within the same organellar compartment (Sections 4.3.1 and 4.3.2) demonstrates that both thermodynamic regimes operate under the same physicochemical constraints; high stromal Mg^2+^, extreme macromolecular crowding, thylakoid pH gradients. Chloroplasts thus offer a natural system for testing how environmental parameters modulate phase boundaries in vivo, complementing the in vitro reconstitution approaches that currently dominate the field.

Systems‐level condensate integration. The *FLC* locus alone is regulated by at least four condensate systems with distinct thermodynamic behaviors, FRI (UCST), GI/FKF1 (temperature‐dependent dissolution), FCA/FLL2 (RNA‐dependent percolation), and DCP5/SSF (chromatin‐anchored) (Section 4.1.4). Whether these systems operate independently, competitively, or cooperatively at the same locus, and how cells resolve potentially conflicting condensate signals, represents a fundamental question. Multi‐omics approaches combining condensate‐specific proteomics, single‐molecule imaging, and metabolomics will be needed to map interaction networks across condensate types and understand how cells orchestrate their collective function.

Taken together, the systems reviewed here establish that plant condensate biology is consistent with physicochemical principles, including Flory–Huggins‐type thermodynamics, UCST/LCST phase behavior, sticker‐and‐spacer sequence grammar that are encoded in protein sequence features. These properties are further tuned by environmental inputs, through defined molecular mechanisms (redox chemistry, PTMs, photoreceptor switching), and have been further shaped by natural selection for ecological adaptation. The challenge ahead is to move from cataloguing individual condensate systems to understanding the rules by which cells deploy, regulate, and integrate them as a coherent regulatory network.

## Funding

This work was supported by grants from Horizon Europe European Research Council (10.13039/501100000781) * 101126019 to P.N.M. and European Union and Greek national funds through the Operational Program Competitiveness, Entrepreneurship, and Innovation * T2ΕΔΚ–00597 under the call RESEARCH–CREATE–INNOVATE “BIOME” to P.N.M. Work in the Staiger lab is supported by the German Research Foundation (10.13039/501100001659) * STA653/14‐1 to D.S. Views and opinions expressed are those of the authors only and do not necessarily reflect those of the European Union or the European Research Council Executive Agency. Neither the European Union nor the granting authority can be held responsible for them.

## Conflicts of Interest

The authors declare no conflicting interests.

## Data Availability

The review does not contain original research data generated by the authors.
